# Systematic review and meta-analysis of surgical drain management after the diagnosis of postoperative pancreatic fistula after pancreaticoduodenectomy: draining-tract-targeted works better than standard management

**DOI:** 10.1007/s00423-020-02005-8

**Published:** 2020-10-26

**Authors:** Sergio Pedrazzoli, Alessandra R. Brazzale

**Affiliations:** 1 Università degli Studi di Padova, Via Crescini, 39, 35126 Padua, Italy; 2 Dipartimento di Scienze Statistiche, Università degli Studi di Padova, Padua, Italy

**Keywords:** Pancreas, Pancreatic surgery, Pancreaticoduodenectomy, Duodenopancreatectomy, Surgical drains, Pancreatic fistula

## Abstract

**Purpose:**

Drains’ role after pancreaticoduodenectomy (PD) is debated by proponents of no drain, draining selected cases, and early drain removal. The aim of the study was to assess the effect of “standard” and “draining-tract-targeted” management of abdominal drains still in situ after diagnosing a postoperative pancreatic fistula (POPF).

**Methods:**

PubMed and Scopus were searched for “pancreaticoduodenectomy or pancreatoduodenectomy or duodenopancreatectomy,” “Whipple,” “proximal pancreatectomy,” “pylorus-preserving pancreatectomy,” and “postoperative pancreatic fistula or POPF.”. Main outcomes included clinically relevant (CR) POPF, grade-C POPF, overall mortality, POPF-related mortality, and CR-POPF-related mortality. Secondary outcomes were incidence of radiological and/or endoscopic interventions, reoperations, and completion pancreatectomies.

**Results:**

Overall, 12,089 studies were retrieved by the search of the English literature (01/01/1990–31/12/2018). Three hundred and twenty-six studies (90,321 patients) reporting ≥ 100 PDs and ≥ 10 PD/year were finally included into the study. Average incidences were obtained by averaging the incidence rates reported in the single articles. Pooled incidences were calculated by combining the number of events and the total number of patients considered in the various studies. These were then meta-analyzed using DerSimonian and Laird’s (1986) method. Pearson’s chi-squared test was used to compare pooled incidences between groups. Post hoc testing was used to see which groups differed. The meta-analyzed incidences were compared using a fixed effect for moderators. “Draining-tract-targeted” management showed a significant advantage over “standard” management in four clinically relevant outcomes out of eight according to pool analysis and in one of them according to meta-analysis.

**Conclusion:**

Clinically, “draining-targeted” management of POPF should be preferred to “standard” management.

**Electronic supplementary material:**

The online version of this article (10.1007/s00423-020-02005-8) contains supplementary material, which is available to authorized users.

## Introduction

Pancreatoduodenectomy (PD) has become safer over the past two decades, but POPF and its severe complications are still responsible for a significant perioperative mortality rate (approximately 1%) and quite a high morbidity rate (66–73%) [1–3].

Several different approaches have been used in efforts to mitigate the impact of POPFs, like different variants of pancreatic anastomosis [4–6], the use of fibrin and acrylic glues [7, 8] of the hormone somatostatin, or its synthetic analogs (SA) [9, 10] of internal or external stents [2, 11–13]. Placing drains during pancreatic surgery is a common strategy for preventing fluid accumulations and their infection, to mitigate POPF-related complications and to facilitate the detection of other intra-abdominal complications, including hemorrhage [14, 15]. But abdominal drains can be responsible for the retrograde infection, hollow viscus decubitus, pain, discomfort, foreign body reaction, and prolonged hospital stays [16]. Drains generate considerable negative pressure potentially responsible for the formation of POPFs [17]. Debate on the real usefulness of surgical drains was triggered by Jeekel [18] in 1992. Subsequent prospective randomized trials designed to compare patients with and without drains after pancreatic surgery produced contradictory findings [19, 20]. Several systematic reviews and meta-analyses have since been performed on this issue [21–30]. Unfortunately, the level of evidence for all the above-mentioned studies was moderate, low, or very low [29, 30].

Prophylactic drain placement during surgery is part of the “standard” management in order to avoid POPF [31]. Unfortunately, even with drains, the clinical burden of a POPF may be significant.

An important advance for the purposes of assessing the efficacy of different mitigating procedures on the number and severity of POPFs came with the international classification of fistulae [32, 33]. Early recognition of a POPF in its harmless state of biochemical leak might help to reduce the risk of subsequent life-threatening complications [34].

**Fig. 1:**
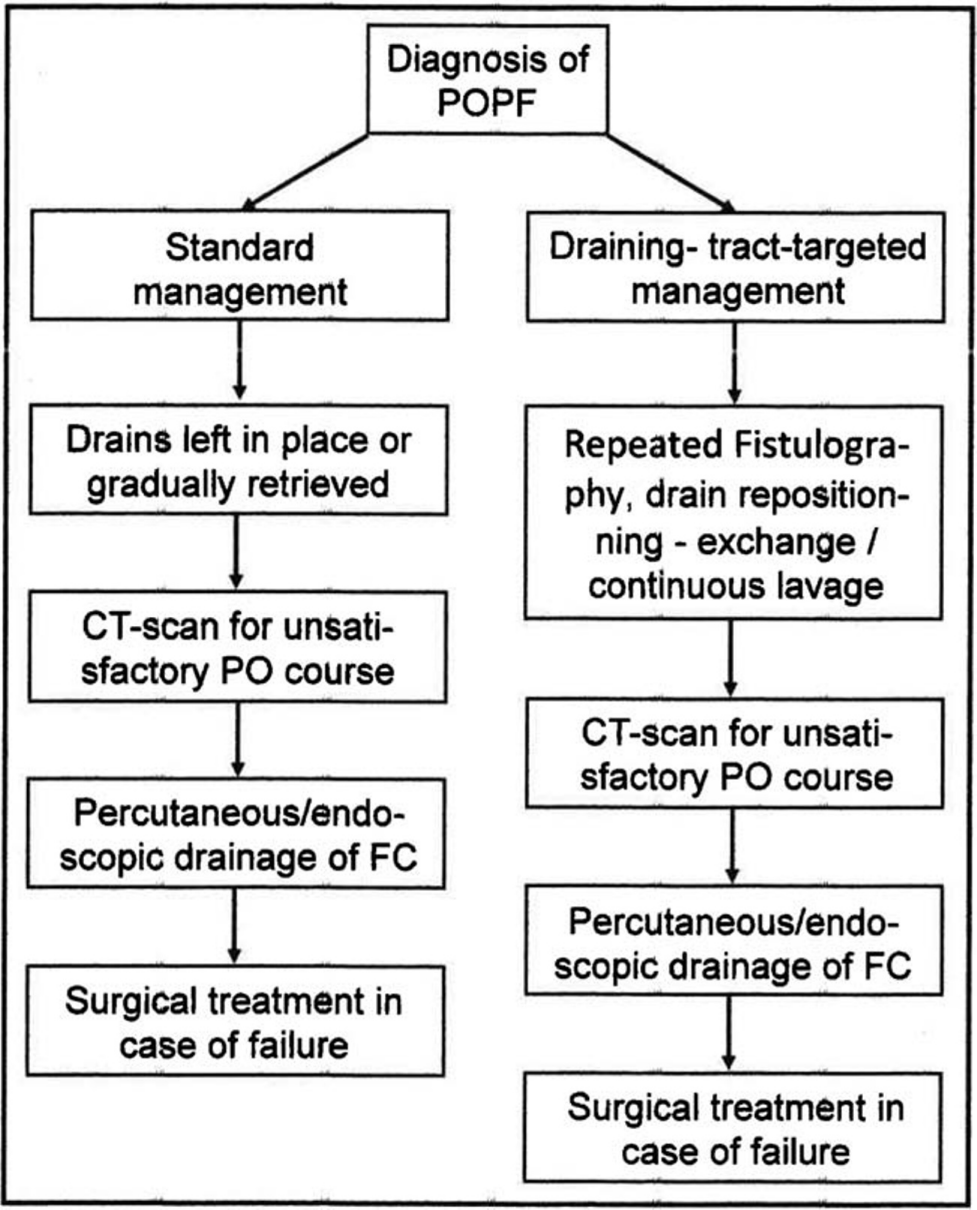
Flow chart of the management of a POPF according to “standard” and “draining-tract-targeted” management of surgical drains

The “standard” management of a POPF included maintaining drains in situ or gradually withdrawn and any further treatment was started only after there was a documented fluid collection and/or abscess. First-line management involved non-surgical options (percutaneous and/or endoscopic and/or endovascular treatment) whenever possible, followed by surgical treatment in the event of failure (Fig. 1) [35, 36]. None of the studies included in the previously mentioned systematic reviews examined the feasibility of a different approach to drain management, after a POPF has been diagnosed, to prevent it from developing into a CR-POPF and causing potentially life-threatening complications.

A “draining-tract-targeted” management of a POPF was proposed by some authors [37–39] and included starting the management as soon as possible after the diagnosis of a POPF to try preventing its harmful evolution. The drain’s path was used to study the fistula by means of a fistulography, to drain any fluid collections and, possibly, to perform a continuous washing of the fistula (Fig. 1).

The present review was aimed at assessing all the different approaches for managing surgical drains after a POPF has been diagnosed, in an effort to identify the best option (if any), capable of reducing the impact of POPFs on patients’ postoperative course [38–40].

## Methods

A previously considered corpus of 208 studies published from January 1, 1990, to December 31, 2015, on the morbidity and mortality rates after pancreaticoduodenectomy in 60,739 patients [2] was retrieved. Then, a comprehensive, systematic literature search was run in PubMed (Medline) and Scopus to identify further studies published from January 1, 2016, to December 31, 2018 (including articles published electronically ahead of print). The search terms were “pancreaticoduodenectomy or pancreatoduodenectomy or duodenopancreatectomy,” “Whipple,” “proximal pancreatectomy,” “pylorus-preserving pancreatectomy,” and “postoperative pancreatic fistula or POPF.” Additional references were sought by cross-checking the bibliographies of the full-text articles selected according to the inclusion criteria. All causes for a proximal pancreatectomy, for both malignant and benign diseases, were included. Series containing only cases of chronic pancreatitis and/or trauma were excluded.

### Inclusion and exclusion criteria

Published studies were included if (1) they were case-control studies, cohort studies, or randomized controlled trials (RCTs) published in the English language in peer-reviewed journals; (2) clearly defined pathology procedures (for benign or malignant pancreatic lesions), and surgical procedures had been used; and (3) they included at least 100 PDs performed at centers handling at least 10 procedures a year, to avoid any bias associated with limited experience. Although a minimum of 10 PDs/year is no longer enough to define a center as “high-volume” [3], this threshold was retained to avoid discrepancies between the two periods considered in the review (1990–2015 and 2016–2018).

Publications were excluded if (1) they failed to meet any of the above-mentioned criteria; (2) they involved studies reporting partially or wholly duplicated data on patients described in previously published works; (3) they concerned studies focusing exclusively on laparoscopic surgery; or (4) they were reviews, editorials, expert opinions, case reports, or letters to the editor, not containing the authors’ data.

### Statistical analysis

Averaged incidences were obtained by averaging the incidence rates reported in the single articles. Pooled incidences were calculated by combining the number of events and the total number of patients considered in the various studies. Pearson’s chi-squared test was used to compare incidences between groups. Post hoc testing based on Tukey contrasts was used to see which groups differed from each other. Averaged incidences were furthermore meta-analyzed using a random effects model according to DerSimonian and Laird’s (1986) method. Differences between the summary estimates were tested using a fixed effects moderator model. A significance level of 0.05 was set, below which *P* values were considered statistically significant. All statistical analyses were carried out using R version 3.6.2 (2019-12-12)—copyright (C) 2019, the R Foundation for Statistical Computing. In particular, the metafor package was used for meta-analysis.

### Analysis of postoperative drain management

Unfortunately, the quality of reporting on PO drain management and perioperative data varied considerably in the publications considered, so they were grouped on the grounds of completeness of reporting on POPF diagnosis and grading [32, 33], management of PO abdominal drains up until their removal, and percutaneous, endoscopic, or surgical management of POPF-related complications.

This led to the following groups and *subgroups.*

- *Group A* included studies adequately reporting (i) details of the POPF diagnostic criteria [32, 33], and drain management after the POPF was diagnosed; (ii) details of percutaneous/endoscopic drainage and/or surgical management of POPF-related complications, including completion pancreatectomies; in patients in *subgroup A1* (“standard” drain management), drains were left in place, or gradually retrieved, until resolution of POPF; in patients in *subgroup A2* (“draining-tract-targeted” management), drains were replaced under fluoroscopic control and/or treated with lavage.
- *Group B* included studies inadequately reporting the details listed in the above item (ii), and patients were divided into *subgroup B1* and *subgroup B2* according to their drain management, as above.
- *Group C* included studies inadequately reporting the details listed in both the above items (i) and (ii).

When considering the characteristics of the studies and the main outcomes, *subgroups A1* and *B1* were then pooled together in *group A1-B1*, and *subgroups A2* and *B2* were pooled together in *group A2-B2*, while only *subgroups A1* and *A2* (the only ones with adequate data) were considered in terms of the secondary outcomes.

### Main outcomes and measures

The main outcomes were CR-POPF rate (i.e., POPFs graded as B/C), grade-C POPFs, overall PO mortality rate, overall POPF-related mortality rate, and grade B/C POPF mortality rate. For studies published before the publication of ISGPF declarations [32], all symptomatic fistulas were considered CR-POPF to compare the results of all CR-POPF thus obtained with those diagnosed only according to ISGPF criteria [32]. Secondary outcomes concerned the incidence of radiological and/or endoscopic interventions, reoperations, and completion pancreatectomies.

## Results

**Fig. 2:**
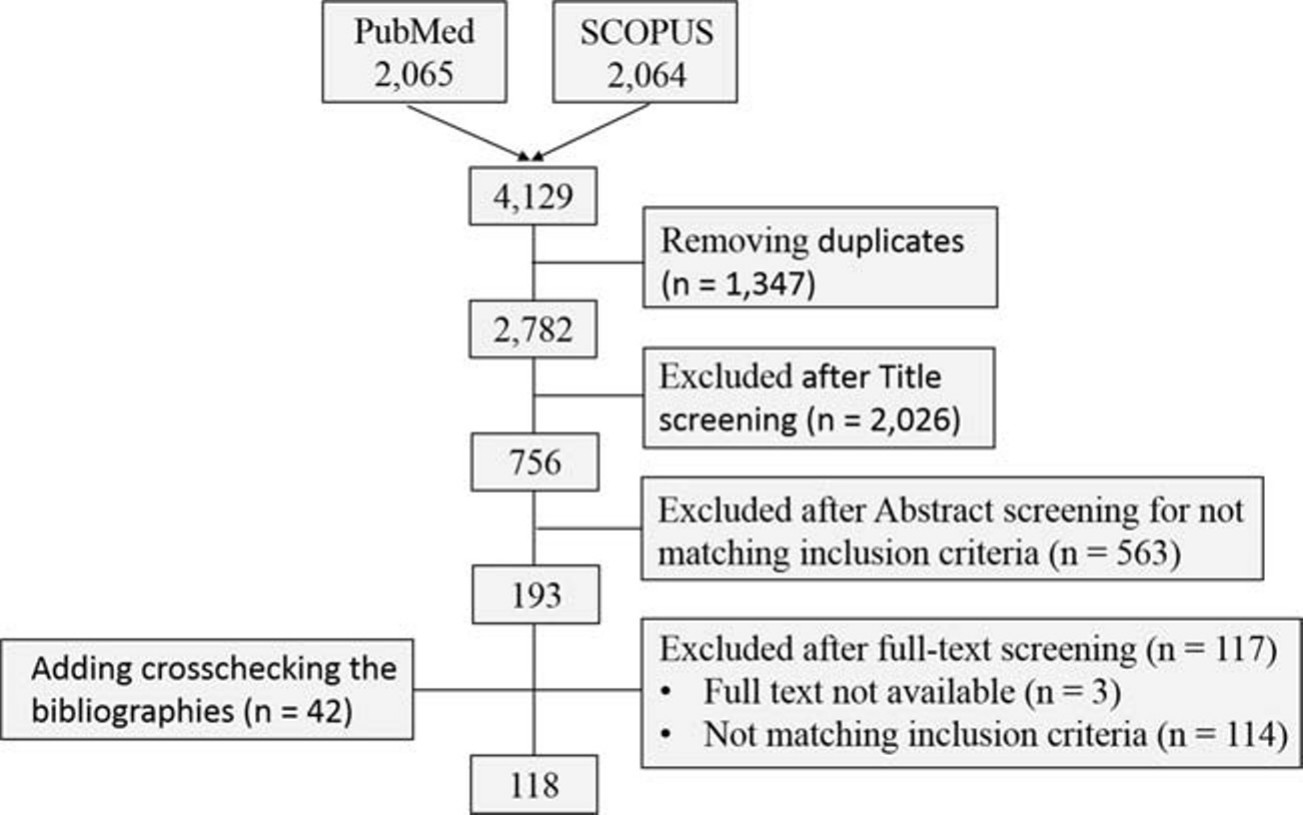
Flow chart of study selection in a systematic review

In all, 4129 studies were retrieved from the major databases. After removing 1347 duplicates and excluding 2589 studies that did not meet our inclusion criteria, the full texts of the remaining 193 studies were retrieved. An additional 42 studies were identified by cross-checking the bibliographies of these 193 full-text articles. Then, 114 studies were omitted because they were inconsistent with our inclusion criteria and another 3 because the full texts were unavailable (Fig. 2). The remaining 118 studies, together with 208 studies selected previously, [2] gave us a population of 90,321 patients considered in the present review.

**Fig. 3:**
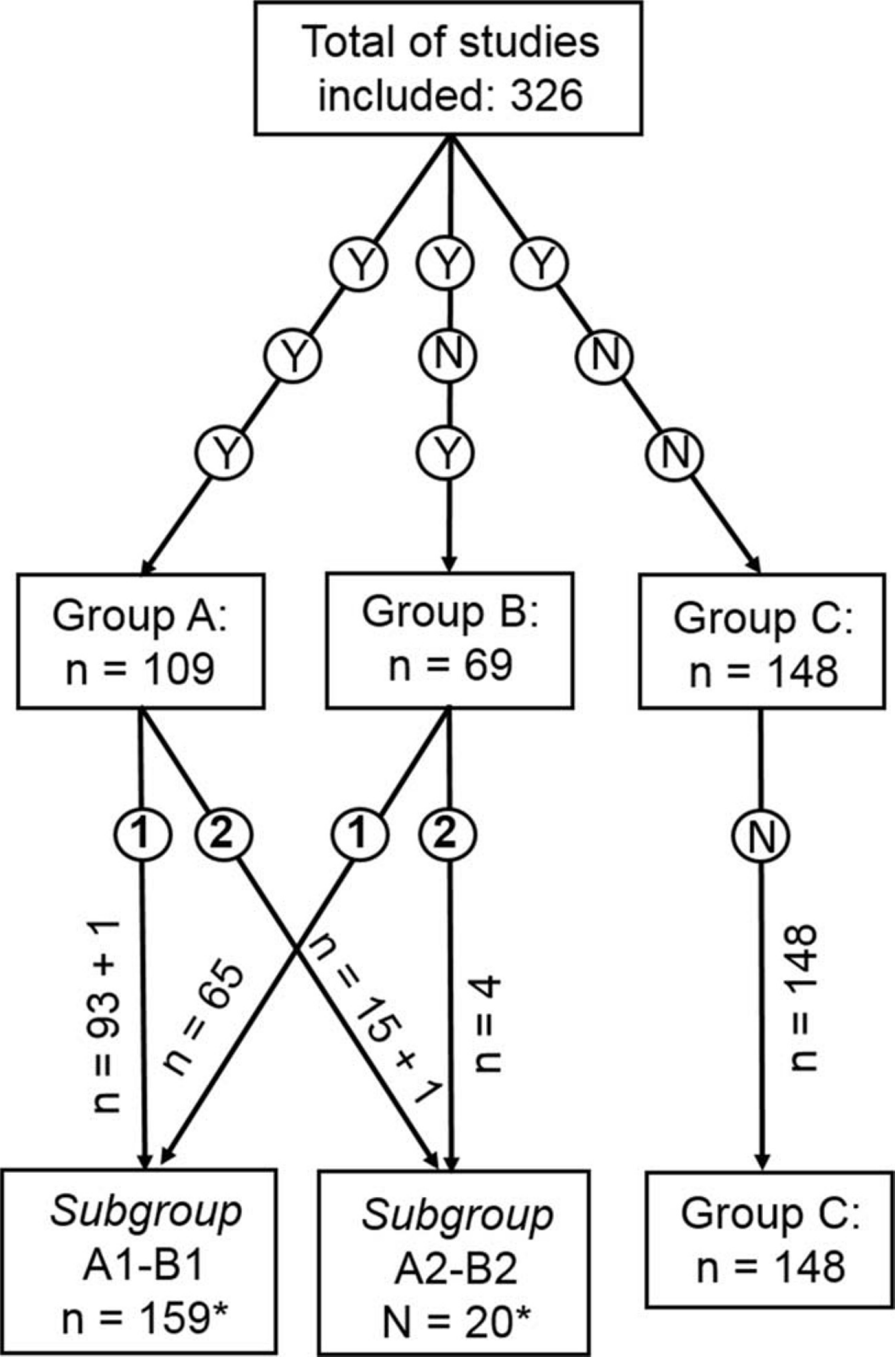
Flow chart of study division into groups and subgroups in relation to the adequacy of the information on diagnostic criteria of POPF, abdominal drains management after the diagnosis of POPF, percutaneous/endoscopic drainage, and/or surgical management of complications related to POPF completion pancreatectomies included. *Patients in one group A study were shared between subgroups A1 and A2 depending on their postoperative surgical drain management

The studies were then divided into three groups according to our previously explained criteria (group A1-B1; group A2-B2; group C; Figure 3, Table 1). One of the more recent studies was included in *subgroups A1* and *A2* because it covered the different types of surgical drain management, giving a total of 327 instead of 326 studies. Group A1-B1 included 159 studies (37,489 patients), group A2-B2 included 20 studies (5037 patients), and group C included 148 studies (47,795 patients). A Whipple procedure was reportedly used in 37,170 patients and a pylorus-preserving PD (PPPD) in 21,819, while no data were available for 31,332 patients (34.69%). The distribution of Whipple procedures and PPPD differed significantly between the three groups (Whipple: 57.40%, 73.45%, and 67.06%; PPPD: 42.60%, 26.55%, and 32.94%, respectively; *P* < 0.001). A PJ or PG was performed in 67,573 and 8987 patients, respectively, while no data were available for 13,761 patients (15.24%). The distribution of PJ and PG also differed significantly between the three groups (PJ: 85.22%, 83.86%, and 91.35%; PG: 14.78%, 16.14%, and 8.65%; *P* < 0.001). An internal or external stent was used in 17,026 and 11,780 patients, respectively, while no stent was used in 29,441 patients, and no data were available for 32,074 patients (35.51%). The proportional usage of internal or external stents, or no stents differed significantly between the three groups too (internal: 33.36%, 24.00%, and 26.00%; external: 13.45%, 17.34%, and 27.58%; no stents: 53.19%, 58.66%, and 46.42%: *P* < 0.001). Closed suction or passive drainage types of surgical drain were used in 11,373 and 59,674 patients, respectively; no drain was used in 1029 patients, and no data were available for 18,245 patients (20.20%). The proportions of patients managed with closed suction or passive drainage solutions, or no drains differed significantly between the three groups (closed suction: 16.77%, 45.42%, and 9.73%; passive drainage: 81.54%, 54.58%, and 88.91%; no drain: 1.69%, 0%, and 1.36%; *P* < 0.001). Fibrin glue was used in 2322 patients to strengthen the pancreatico-enteric anastomosis; no glue was used in 48,326 patients, and no data were available for 39,673 patients (43.92%). The distribution of fibrin glue usage between the three groups differed significantly (with glue: 1.79%, 3.66%, and 8.08%; without glue: 98.21%, 96.34%, and 91.92%; *P* < 0.001). Postoperatively, 14,692 patients were given somatostatin or SA, 34,343 were not and no data were available for 41,286 patients (45.71%). The distribution of the perioperative somatostatin or SA administration differed significantly between the three groups (38.90%, 26.69%, and 20.58% were given the hormone; 61.10%, 73.31%, and 79.42% were not; *P* < 0.001).

**Table 1:** Characteristics of groups A1-B1, A2-B2, and C and the sample overall

Characteristics	Group A1-B1	Group A2-B2	Group C	Overall	*P*
Studies (n.)	159^a^	20^a^	148	327^a^	
Patients (n.)	37,489	5037	47,795	90,321	
Mean	235.78	251.85	322.94	276,21	
SD^b^	175.87	136.92	484.48	351,78	
Procedures (n.):					
Whipple	15,873	3231	18,066	37,170	< 0.001
PPPD^c^	11,778	1168	8873	21,819	
Not reported	9838	638	20,856	31,332	
Studies without data	36	1	41	78	
Pancreatic anastomosis (n.)					
PJ^d^	28,151	3787	35,635	67,573	< 0.001
PG^e^	4883	729	3375	8987	
Not reported	4455	521	8785	13,761	
Studies without data	17	2	33	52	
Stents (n.):					
Internal	8959	1148	6919	17,026	< 0.001
External	3613	829	7338	11,780	
None	14,284	2805	12,352	29,441	
Not reported	10,633	255	21,186	32,074	
Studies without data	40	2	55	97	
Drains (n.):					
Closed suction	6100	2288	2985	11,373	< 0.001
Passive drainage	29,656	2749	27,269	59,674	
None	613	0	416	1029	
Not reported	1120	0	17,125	18,245	
Studies without data	6	0	40	46	
Fibrin glue:					
Used	458	128	1736	2322	< 0.001
Not used	25,193	3374	19,759	48,326	
Not reported	11,838	1535	26,300	39,673	
Studies without data	49	6	80	133	
Somatostatin or analogs:					
Used	9341	880	4471	14,692	< 0.001
Not used	14,669	2417	17,257	34,343	
Not reported	13,479	1740	26,067	41,286	
Studies without data	48	6	73	127	

^a^Patients in one study were shared between *subgroups* A1 and A2 depending on their postoperative surgical drain management

^b^Standard deviation

^c^Pylorus-preserving pancreaticoduodenectomy

^d^Pancreaticojejunostomy

^e^Pancreaticogastrostomy

### Main outcomes

The results observed in the three groups of patients, considering for CR-POPFs, only those studies reporting ISGPF/ISGPS classifications [32, 33], are given in Table 2. The overall incidence of CR-POPFs was reported in 237 studies (72.48%) describing 63,921 of 90,321 patients (70.77%). The pooled incidence of CR-POPFs was 13.33% and was significantly higher in group A2-B2 (*P* = 0.009), while the DL incidence was 12.56% without any significant difference between the three groups (*P* = 0.498). The overall incidence of grade-C POPFs was only reported by 193 studies (59.02%) describing 54,241 patients (60.05%). Their pooled incidence was 3.45% and was significantly lower in group A2-B2 than in groups A1-B1 and C (*P* = 0.001); the DL incidence was 3.27% and was lower in group A2-B2 than in groups A1-B1 and C but the difference was not significant (*P* = 0.091). The overall PO mortality rate was reported in 296 studies (90.52%) describing 76,743 patients (84.97%). The pooled incidence was 2.54% and was significantly higher for group C (*P* = 0.007). The DL incidence was 2.54% without any significant difference between the three groups (*P* = 0.187). The overall POPF-related mortality was reported in 268 studies (81.96%) describing 74,437 patients (82.41%). The pooled POPF-related mortality was 1.06% and was significantly lower in group A2-B2 than in groups A1-B1 and C (*P* = 0.004). The DL incidence of POPF-related mortality was 1.23% and was lower in group A2-B2 than in groups A1-B1 and C but the difference was not significant (*P* = 0.088). The CR-POPF-related mortality was reported in 180 studies (55.05%) reporting 6594 CR-POPFs among 49,319 patients (13.37%). The pooled incidence was 7.28% and the DL incidence was 8.88%; the lowest incidence found for group A2-B2 was not statistically significant (*P* = 0.083 and = 0.245, respectively).

**Table 2:** Results for primary endpoints in groups A1-B1, A2-B2, and C, and the sample overall

Primary endpoints	Group A1-B1	Group A2-B2	Group C	Overall	*P*
CR-POPF^a^					
Studies	119/159	16/20	102/148	237/327^d^	
Patients	25,794	3663	34,464	63,921	
Mean	216.76	228.94	337.88	269.71	
SD^b^	154.99	126.99	500.61	351.88	
Average incidence (%)	13.38	14.24	14.35	13.90	
SD^b^	8.21	10.89	8.18	8.38	
Pooled incidence (%)	12.91	14.50	13.53	13.33	= 0.009
DL incidence^c^ (%)	12.08	13.95	13.18	12.56	= 0.498
Grade-C POPF					
Studies	103/159	14/20	76/148	193/327^d^	
Patients	23,264	3622	27,355	54,241	
Mean	225.86	258.71	359.93	281.04	
SD^b^	160.58	132.94	561.25	376.93	
Average incidence (%)	3.77	2.36	3.91	3.72	
SD^b^	4.61	2.39	3.16	3.97	
Pooled incidence (%)	3.63	2.15	3.48	3.45	= 0.001
DL incidence^c^ (%)	3.18	2.11	3.58	3.27	= 0.091
Overall PO mortality					
Studies	148/159	20/20	128/148	296/327^d^	
Patients	33,795	5037	37,911	76,743	
Mean	228.35	251.85	296.18	259.27	
SD^b^	174.44	136.92	427.85	310.25	
Average incidence (%)	2.26	2.51	2.74	2.49	
SD^b^	2.11	2.24	2.66	2.37	
Pooled incidence (%)	2.37	2.30	2.72	2.54	= 0.007
DL incidence^c^ (%)	2.33	2.49	2.78	2.54	= 0.187
POPF-related mortality					
Studies	147/159	18/20	103/148	268/327^d^	
Patients	34,098	4762	35,577	74,437	
Mean	231.96	264.56	345.41	277.75	
SD^b^	173.20	138.54	563.65	376.73	
Average incidence (%)	1.03	0.49	1.21	1.07	
SD^b^	1.37	0.63	1.49	1.39	
Pooled incidence (%)	1.04	0.61	1.14	1.06	= 0.004
DL incidence^c^ (%)	1.25	0.79	1.30	1.23	= 0.088
CR-POPF^a^-related mortality					
Studies	108/159	14/20	60/148	180/327^d^	
Patients with CR-POPF	2891	501	3202	6594	
Mean	26.77	35.79	51.65	35.84	
SD^b^	25.27	35.29	71.45	47.93	
Average incidence (%)	8.27	5.00	9.13	8.31	
SD^b^	11.42	7.84	11.98	11.38	
Pooled incidence (%)	7.47	4.79	7.50	7.28	= 0.083
DL incidence^c^ (%)	9.73	5.65	8.45	8.88	= 0.245

^a^For CR-POPFs, only those studies reporting ISGPF/ISGPS classifications [32, 33] were considered

^b^Standard deviation

^c^DL incidence (%): DerSimonian-Laird estimator

^d^Patients in one study were shared between *subgroups* A1 and A2 depending on their postoperative surgical drain management

If we consider all patients with CR-POPF (both those defined on the basis of the ISGPF/ISGPS classifications [32, 33] and the symptomatic ones reported in studies published before the publication of the ISGPF criteria), the results were similar to those reported in Table 2 for the pooled incidence and DL incidence of CR-POPFs (*P* = 0.009 and 0.699, respectively) while for the CR-POPF-related mortality, the lowest incidence detected for the A2-B2 group became statistically significant for the pooled incidence and remained not significant for the DL incidence (*P* = 0.031 and 0.090, respectively) (see Table 4 in the electronic supplemental material).

### Secondary outcomes

The results of group A studies are reported in Table 3. Concerning the overall incidence of radiological/endoscopic interventions, which was reported in 96/109 group A studies (88.07%), the pooled incidence was 4.14% and the DL incidence was 3.82%, without any significant differences between the two *subgroups* A1 and A2 (*P* = 0.058 and 0.123, respectively). The overall reoperation rate was reported in 108/109 group A studies (99.08%). The pooled incidence of reoperation rate was 3.66% and was significantly lower in *subgroup* A2 than in *subgroup* A1 (*P* < 0.001). The DL incidence was 3.04%, and the lowest incidence found for *subgroup* A2 than in *subgroup* A1 was not statistically significant (0.069%). The overall completion pancreatectomy rate was reported in 99/109 group A studies (90.83%) giving a pooled incidence of 0.54% and a DL incidence of 0.57%. The incidence of completion pancreatectomy was significantly lower in *subgroup* A2 than in *subgroup* A1 according to both the pooled analysis (*P* < 0.001) and to meta-analysis (*P* = 0.004).

**Table 3:** Results for secondary endpoints in group A and *subgroups* A1 and A2

Secondary endpoints	Subgroup A1	Subgroup A2	Group A	*P*
Studies (n.)	93^b^	16^b^	109	
Patients (n.)	22,299	4179	26,478	
Mean	239.77	261.19	242.92	
SD^a^	168.00	136.02	163.31	
Interventions^c^				
Studies	82	14	96	
Patients	19,418	3703	23,121	
Mean	236.80	264.50	240.84	
SD^a^	165.82	134.24	161.27	
Average incidence (%)	4.47	4.61	4.49	
SD^a^	4.66	3.59	4.50	
Pooled incidence (%)	4.03	4.73	4.14	= 0.058
DL incidence^d^ (%)	3.67	3.94	3.82	= 0.123
Reoperations				
Studies	92	16	108	
Patients	22,198	4179	26,377	
Mean	241.28	261.19	244.23	
SD^a^	168.28	136.02	163.49	
Average incidence (%)	3.92	2.32	3.68	
SD^a^	3.78	2.50	3.65	
Pooled incidence (%)	3.93	2.25	3.66	< 0.001
DL incidence^d^ (%)	3.26	2.04	3.04	= 0.069
Completion pancreatectomies				
Studies	83	16	99	
Patients	20,261	4179	24,440	
Mean	244.11	261.19	246.87	
SD^a^	170.67	136.02	165.06	
Average incidence (%)	0.56	0	0.47	
SD^a^	1.22	0	1.14	
Pooled incidence (%)	0.65	0	0.54	< 0.001
DL incidence^d^ (%)	0.67	0.22	0.57	= 0.004

^a^Standard deviation

^b^Patients of one study were divided between *subgroups* A1 and A2 according to the PO management of surgical drains

^c^POPF-related radiological/endoscopic interventions

^d^DL incidence (%): DerSimonian-Laird estimator

Forest plots of the primary and secondary outcomes are reported in the electronic supplemental material.

## Discussion

This comprehensive systematic review pooled analysis and meta-analysis of 326 studies published between January 1, 1990, and December 31, 2018, (referring to 91,321 patients) is the first large review on the management of surgical drains still in situ after a POPF is diagnosed in patients undergoing PD. The main finding was a better result in four clinically relevant outcomes considered (grade-C POPFs; POPF-related mortality rate; reoperations; and completion pancreatectomies) that was statistically significant in all four according to the pooled analysis and only in one (completion pancreatectomies) according to the meta-analysis, in patients who underwent “draining-tract-targeted” (group A2-B2 in Table 2; subgroup A2 in Table 3) than in those undergoing “standard” drain management (groups A1-B1 and C in Table 2; subgroup A1 in Table 3).

Given that an improvement close to significance was also obtained in the three relevant non-significant outcomes at the meta-analysis (grade-C POPF: *P* = 0.091; POPF-related mortality: *P* = 0.088; reoperations: *P* = 0.069; Tables 2 and 3) and that the large and very heterogeneous number of studies included in this review is better analyzed by an aggregate analysis than by a meta-analysis, we believe the improvement of the four outcomes observed in patients treated with “draining-tract-targeted” management is clinically relevant.

The increase in CR-POPF and the decrease in overall PO mortality in patients undergoing “drainage-targeted” management (significant only according to the pooled analysis: *P* = 0.009 and = 0.007) were not included among the clinically relevant outcomes due to the frankly negative results of the meta-analysis (Table 2). The lower pooled and DL incidence of CR-POPF-related mortality in patients undergoing “draining-tract-targeted” management was not significant; the result could be explained by the smaller number of studies reporting this information (180/327 only, Table 2). Finally, there were no significant differences in the pooled and DL incidence of interventions between the three groups of patients (Table 3) confirming that “draining-tract-targeted” management does not exclude the possibility of subsequent interventions (Fig. 1).

These interesting results, although not univocal, emerged despite the relatively small number of studies in group A2-B2 compared with those in groups A1-B1 and C, and despite the lack of homogeneity in the “draining-tract-targeted” management adopted for group A2-B2 [37–39]. Furthermore, comparing the results of the CR-POPFs defined on the basis of the ISGPF/ISGPS classifications [32, 33] (Table 2) with those of CR-POPFs which also included the symptomatic ones reported prior to publication of the ISGPF criteria, the results of the pooled and DL incidence of CR-POPF (*P* = 0.009 and *P* = 0.923) were similar, while the lowest incidence of CR-POPF-related mortality of group A2-B2 than groups A1-B1 and C became statistically significant for the pooled incidence (*P* = 0.031) and remained not significant for the DL incidence (*P* = 0.261) (see Table 4 in the electronic supplemental material).

During the 28 years of this review, only 179/326 studies (54.91%) reported the surgical drain management after diagnosing a POPF with a prevalent use of “standard” drain management (159/179 studies, 88.83%), compared to “draining-tract-targeted” management (20 studies, 11.17%). In the former “standard” case, the drain was left in place or gradually withdrawn, and any further treatment was started only after a CR-POPF was diagnosed and there was a documented fluid collection and/or abscess (Fig. 1). [34, 41–44] In the “draining-tract-targeted” management of POPFs, treatment was started as soon as possible after diagnosing a POPF to reduce the risk of subsequent life-threatening complications (Fig. 1) [34]. “Draining-tract-targeted” management included three different approaches: (1) fistulography through the surgical drains with their subsequent replacement, or repositioning, over a wire [37]; (2) drain replacement with some pigtail or malecot 8–10 Fr one of which was inserted as soon as possible through the fistula into the gastrointestinal lumen [38, 45, 46]; (3) drain replacement under fluoroscopic control and closed lavage with 500 to 3000 mL of natural saline depending on the amylase level in the drained fluid [39]. It is worth emphasizing that using “draining-tract-targeted” management did not prevent subsequent use of percutaneous and/or endoscopic drainage of any fluid collections or abscesses (Fig. 1) (Table 3); these procedures have technical success rates of 100% and 92–97%, respectively, but clinical success rates of only 67–77% and 59%, respectively. [36, 47, 48] Postoperative infections are a major determinant of outcome after PD [49, 50], and drain lavage and/or replacement can also help to reduce PO intra-abdominal infection. The earlier and easier approach to POPF treatment by “draining-tract-targeted” management can help explain the different outcomes of the two approaches.

According to Tomimaru et al. [51] fistulography findings were significantly associated with POPF healing time. An intra-abdominal drainage tube was changed every 1–2 weeks until the POPF is resolved. A “draining-tract-targeted” management of biochemical leakage (BL) was also proposed by Takeda et al. [52] A fistulography was performed weekly, starting on POD 7. The surgical drain was replaced with an 8-Fr soft drain. The weekly routine fistulography was not considered as an interventional procedure because it had only a diagnostic purpose, whereas any additional fistulography and repositioning of drains due to signs of infection were considered as percutaneous intervention therapy. This distinction poses the problem of how to frame the different “draining-tract-targeted” managements of POPFs [37–39, 51, 52] in the ISGPF/ISGPS classifications [32, 33].

In a previous review [2], we reported a POPF-related mortality rate of around 1%, which had remained unchanged over a 25-year study period. This rate increased significantly—to 1.2% (*P* = 0.007)—during the last 3 years covered by the present review. We can therefore assume that, during the last 28 years, the prevalent treatment of a POPF (“standard” drain management) has not changed the POPF-related mortality rate.

Routinely placing intraperitoneal drains after PD was considered a strategy to mitigate the incidence and effects of POPFs. In our present study, 98.57% of the patients with adequate data available on the use of drains had a drain placed at the end of the surgical procedure, and the rate was the same for the patients reported in studies published in the last 3 years (23,533 out of 23,951 patients; 98.25%). Unfortunately, 46 studies (14.11%) failed to report on the type of drainage treatment after surgery (Table 1), and 22 of them were published in the last 3 years (22/118—18.64%).

After the actual usefulness of abdominal drains was called into question for other abdominal surgeries [53–55] and for pancreatic surgery [18], there has been a great debate among the drainers [20] non-drainers [19, 56], selective drainers according to the Fistula Risk Score [57–59], the early drain removers [16, 60], and selective drainers and early removers [61]. Several systematic reviews and meta-analyses that variously included both RCTs and non-RCTs, both on PD and distal pancreatectomy [21–30], led to different opinions regarding the usefulness of drains after PD. Some judged them demonstrably useful [23, 27, 30], while others found no evidence to confirm as much [28, 29]. Some said they are only useful in selected patients (such as those at high risk of POPF) [25]. Other findings were neutral (drains neither increased nor reduced PO complications and mortality) [21, 22, 24, 26]. When drains were used, their early removal seemed a good idea [21, 24, 29, 30]. Unfortunately, the level of evidence for all the above-mentioned studies was moderate, low, or very low [29, 30].

According to McMillan’s study [61], drains can be safely omitted for one in four patients undergoing PD and removed early in a sizable proportion of cases. Drains would nonetheless remain in place after diagnosing a POPF in at least 20.4% of patients. A similar experience was reported by Trudeau et al. [62]. Drains remained in place in 12.75% of patients, the overall CR-POPF rate was 8.7%, and the overall mortality rate was .8%, almost all in patients with CR-POPFs and surgical drains still in place. Therefore, drains remain in place in a relevant percentage of POPFs, and their best treatment is still undefined.

POPF-related mortality rate was reported only in 268 out of 326 studies (82.21%), but only in 61 out of 118 studies (51.69%) of the more recent period (2016–2018). We believe that POPF-related mortality rate is a very important objective parameter to be evaluated, at least as important as complication rate and hospital stay. Unfortunately, neither the International Study Group on Pancreatic Surgery (ISGPS) [33] nor 23 International Expert Centers in pancreas surgery [3] included an accurate definition of POPF-related mortality rate among the outcome parameters of pancreatic surgery.

An important limitation of our review lies in the retrospective analysis of mainly retrospective studies and the large gaps in the reporting of the data as evidenced in Tables 1, 2, and 3. The number of studies and patients with missing data for each characteristic and outcome is indicated in the tables. Complete datasets for all the variables required in Tables 1 and 2 were only available in 67 of the 326 studies considered in this review (20.55%)—and in none of the 118 studies published between 2016 and 2018 (It is noteworthy that the ISGPF classification was reported in 98% of the latter studies). As for the secondary outcomes, they were not only lacking in all 70 studies in group B and 148 studies in group C, but also missing in 9/93 studies in group A1 and 1/16 in group A2.

Another point to mention is that the clinically better outcome for four out of eight outcomes in group A2-B2 and *subgroup* A2 is important but needs to be confirmed in RCTs because it emerged from retrospective studies without enough detail concerning the distribution between the groups of several non-modifiable risk factors (e.g., age, body mass index, comorbidity, underlying diseases, gland texture, and Wirsung duct size). There is also to consider that three different types of “draining-tract-targeted” management were adopted [37–39].

## Conclusion

In conclusion, this review is focused on the management of drains still in situ when a POPF is diagnosed. Instead of leaving them in place, or gradually withdrawing them (as is usually done in the “standard” management approach), their presence can be usefully exploited both for a diagnostic fistulography, to check for any collections communicating with the drainage tract [38, 48], and for therapeutic purpose, using any readily available method to replace or reposition drains under fluoroscopic control and, where necessary, proceed with continuous lavage of the draining tract [37–39, 47, 48]. Compared with “standard” management, the use of a “draining-tract-targeted” management approach achieved clinically better results for four of the eight outcomes considered here.

## Electronic supplementary material

ESM 1 (PDF 21 kb).

ESM 2 (PDF 18 kb).

ESM 3 (PDF 25 kb).

ESM 4 (PDF 23 kb).

ESM 5 (PDF 18 kb).

ESM 6 (PDF 11 kb).

ESM 7 (PDF 12 kb).

ESM 8 (PDF 11 kb).

ESM 9 (PDF 25 kb).

ESM 10 (PDF 20 kb).

ESM 11 (DOCX 51 kb).
