# Supplementary figures and images for: Systematic review and meta-analysis of surgical drain management after the diagnosis of postoperative pancreatic fistula after pancreaticoduodenectomy: draining-tract-targeted works better than standard management
Source: Langenbecks Arch Surg. 2020 Oct 26;405(8):1219–31. doi: 10.1007/s00423-020-02005-8 (PMC7686010; doi:10.1007/s00423-020-02005-8)

CR-POPF  
(group A1-B1)

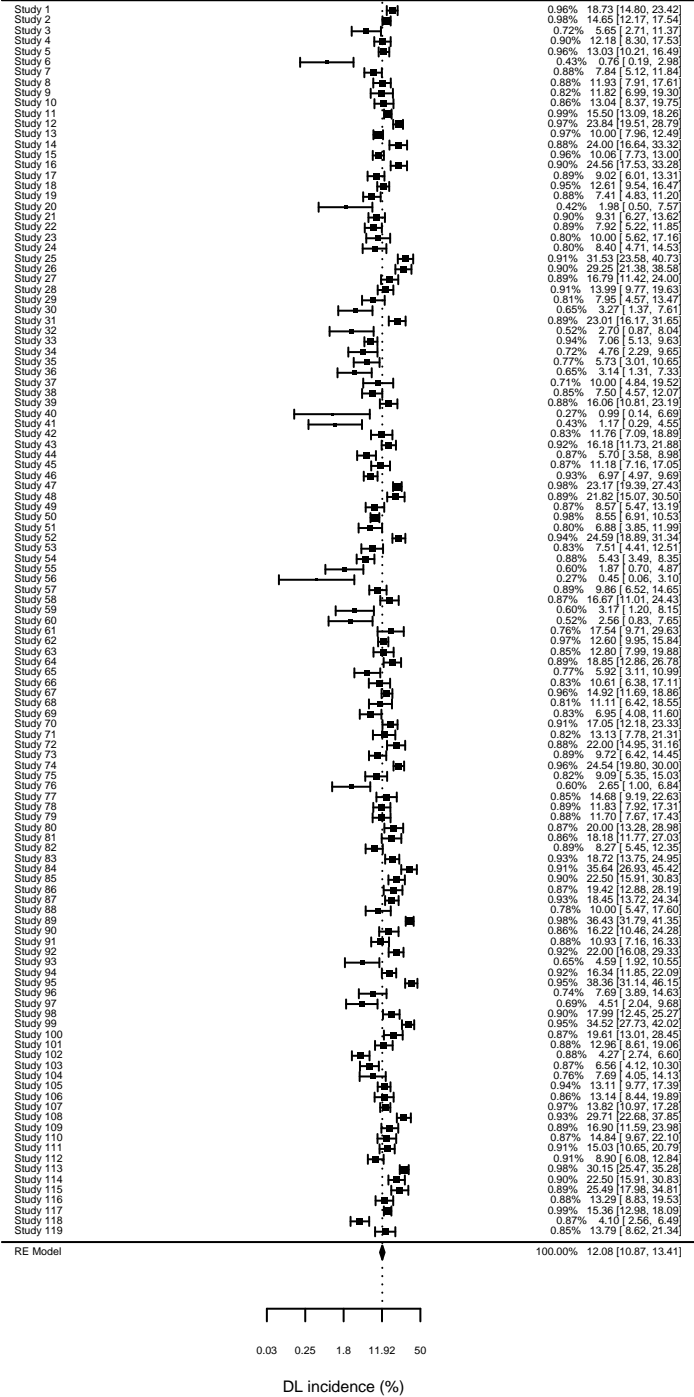

CR-POPF  
(group A2-B2)

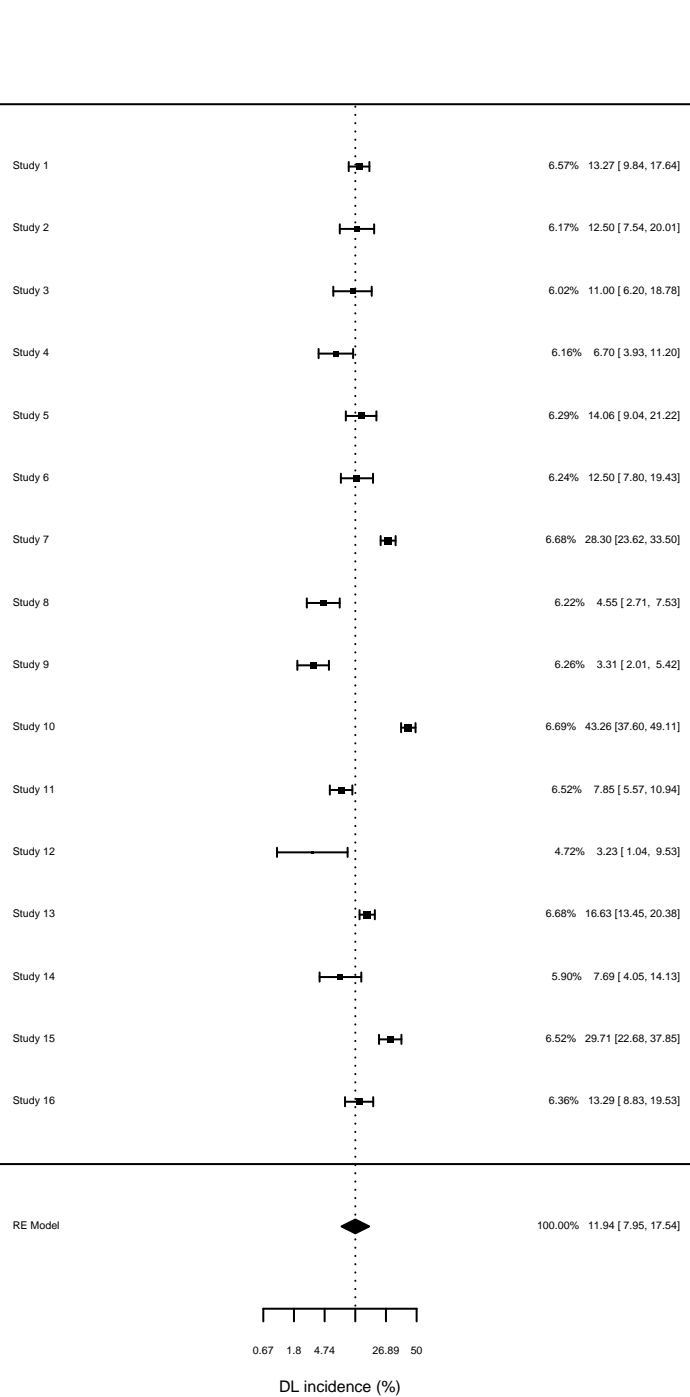

CR-POPF  
(group C)

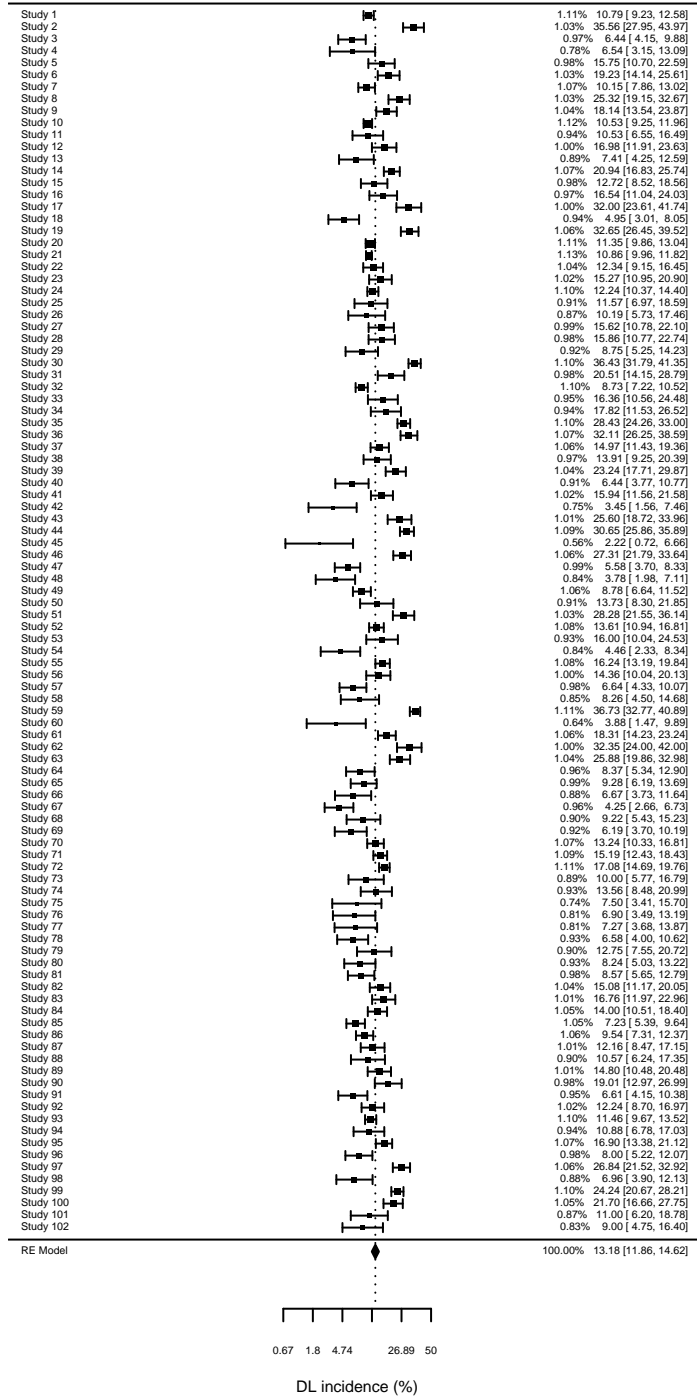

Supplement: Supplementary file 1 — (PDF 21 kb). [file 423_2020_2005_MOESM1_ESM.pdf]

Grade C POPF  
(group A1-B1)

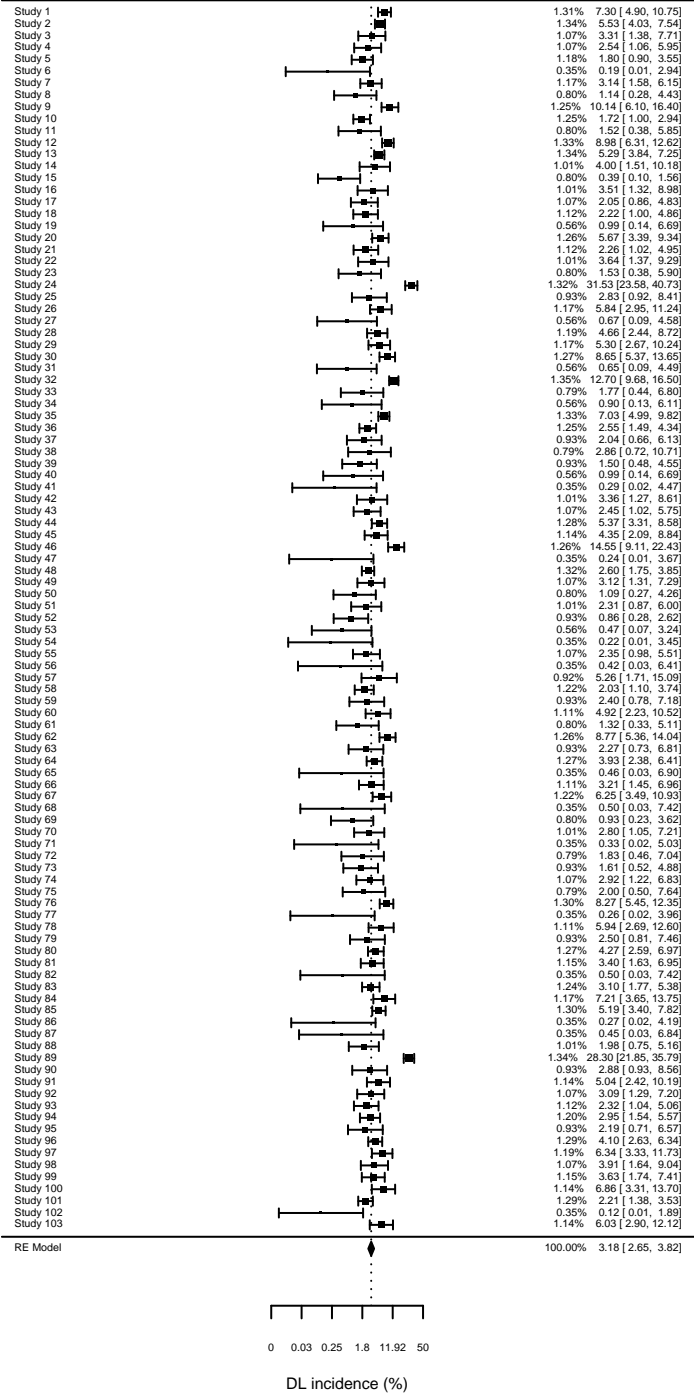

Grade C POPF  
(group A2-B2)

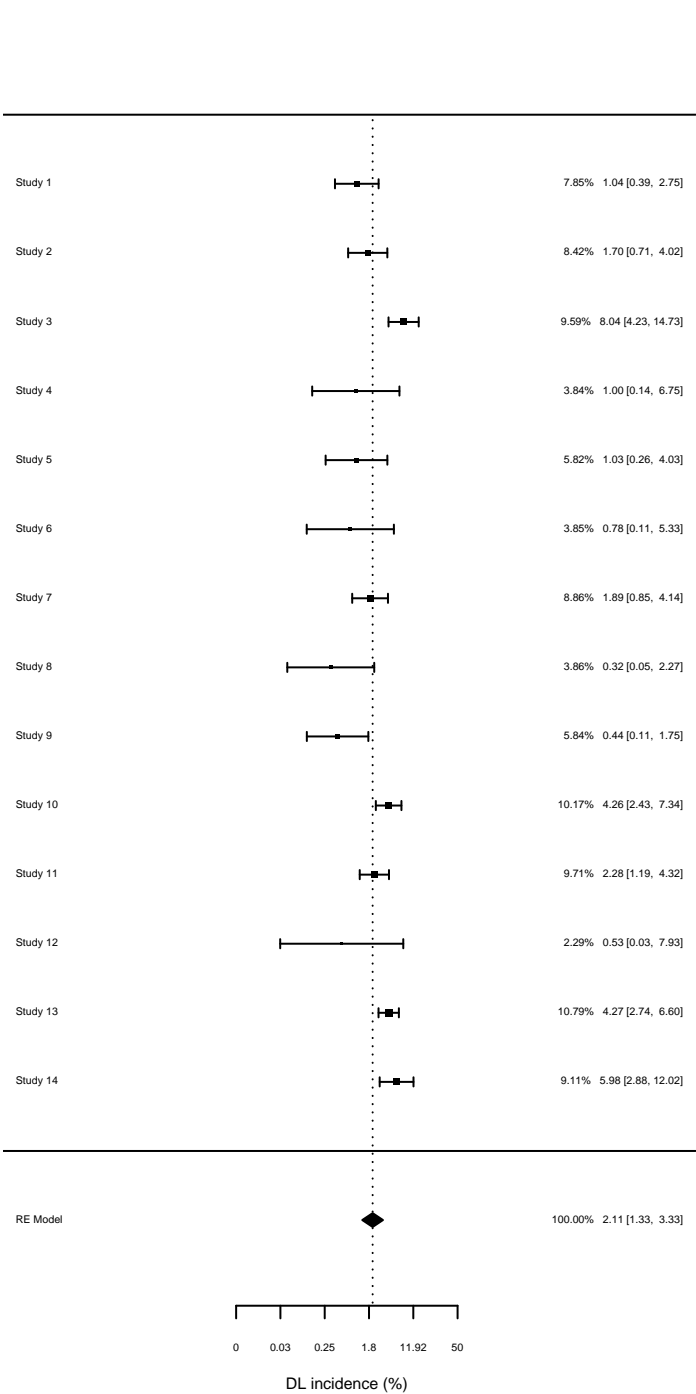

Grade C POPF  
(group C)

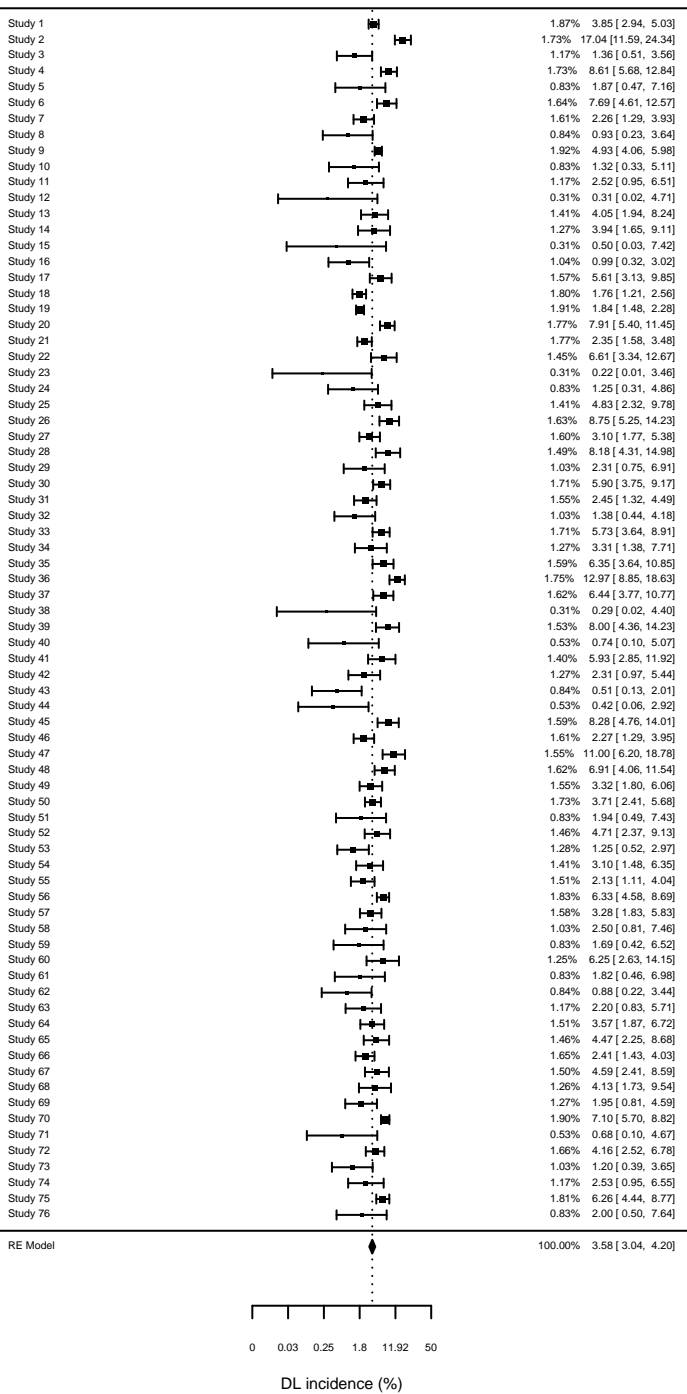

Supplement: Supplementary file 2 — (PDF 18 kb). [file 423_2020_2005_MOESM2_ESM.pdf]

Overall POMR  
(group A1-B1)

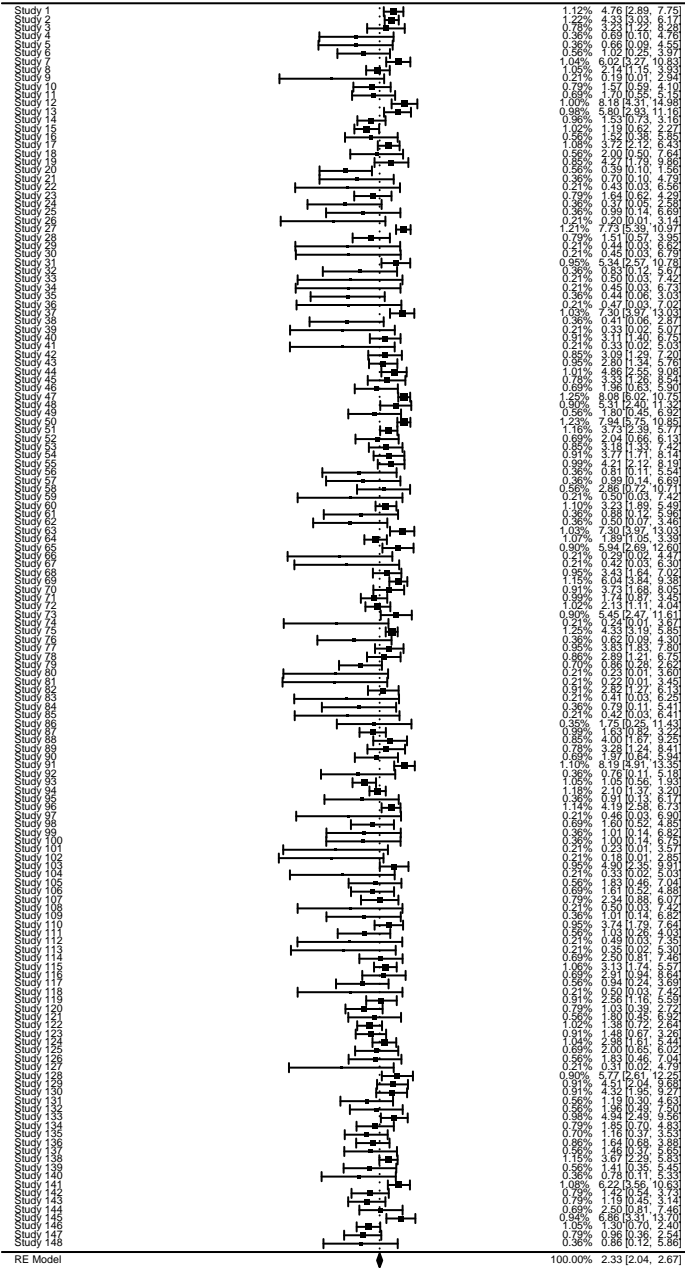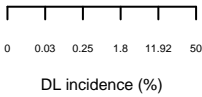

Overall POMR  
(group A2-B2)

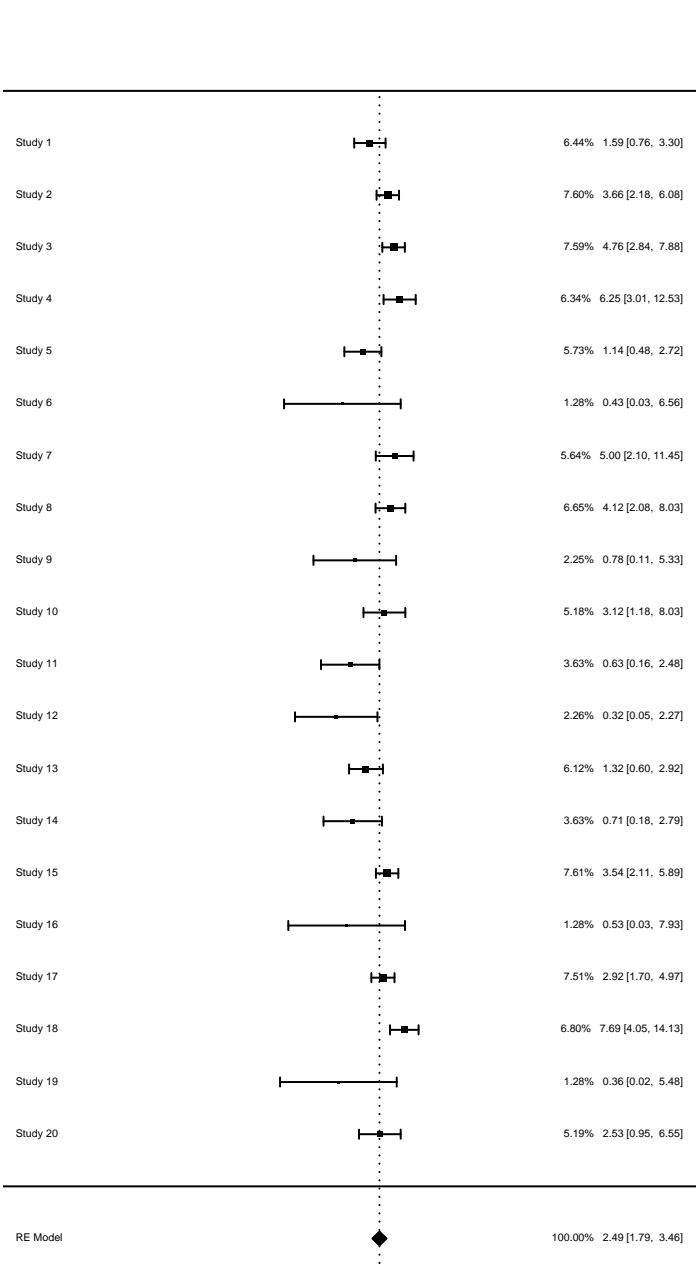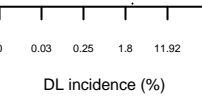

Overall POMR  
(group C)

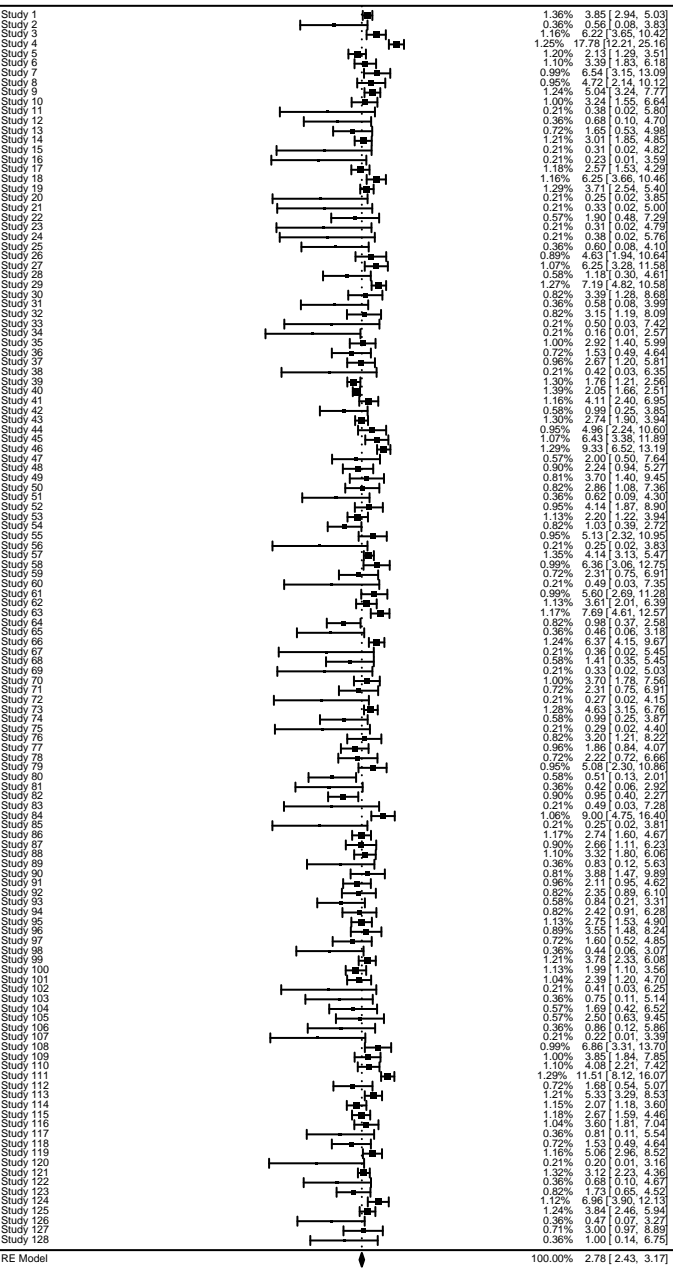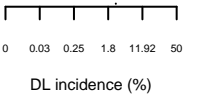

Supplement: Supplementary file 3 — (PDF 25 kb). [file 423_2020_2005_MOESM3_ESM.pdf]

POPF related POMR  
(group A1-B1)

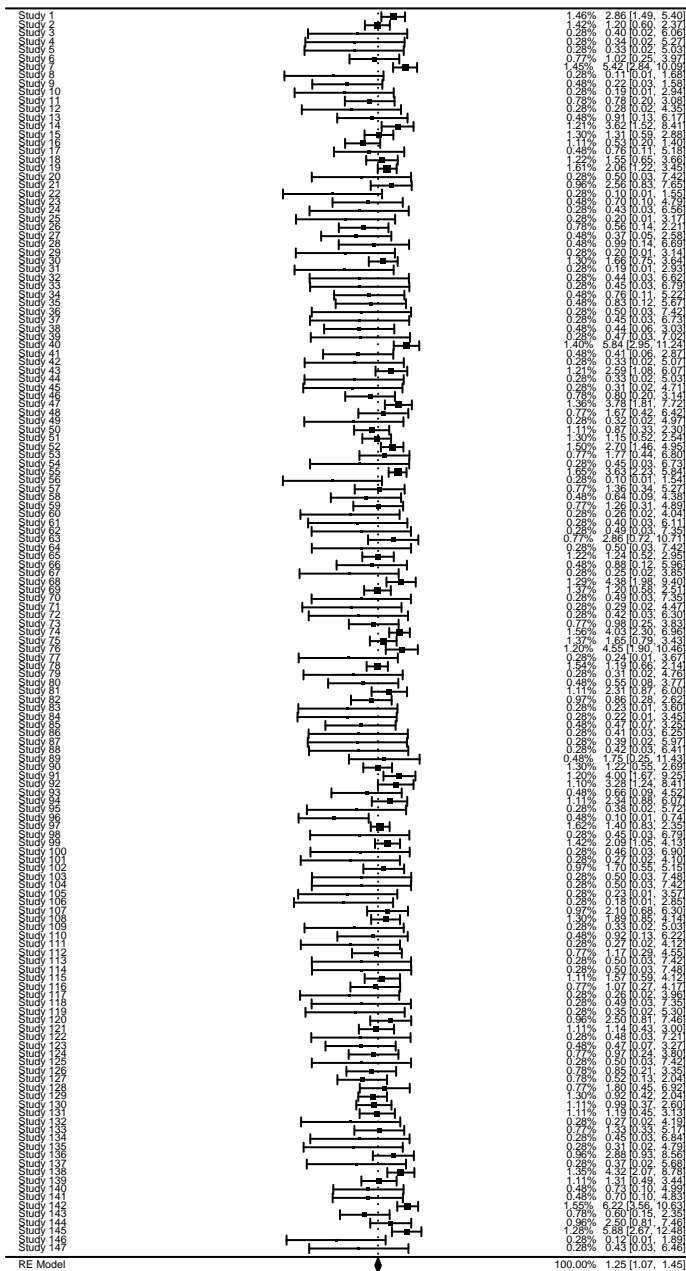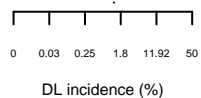

POPF related POMR  
(group A2-B2)

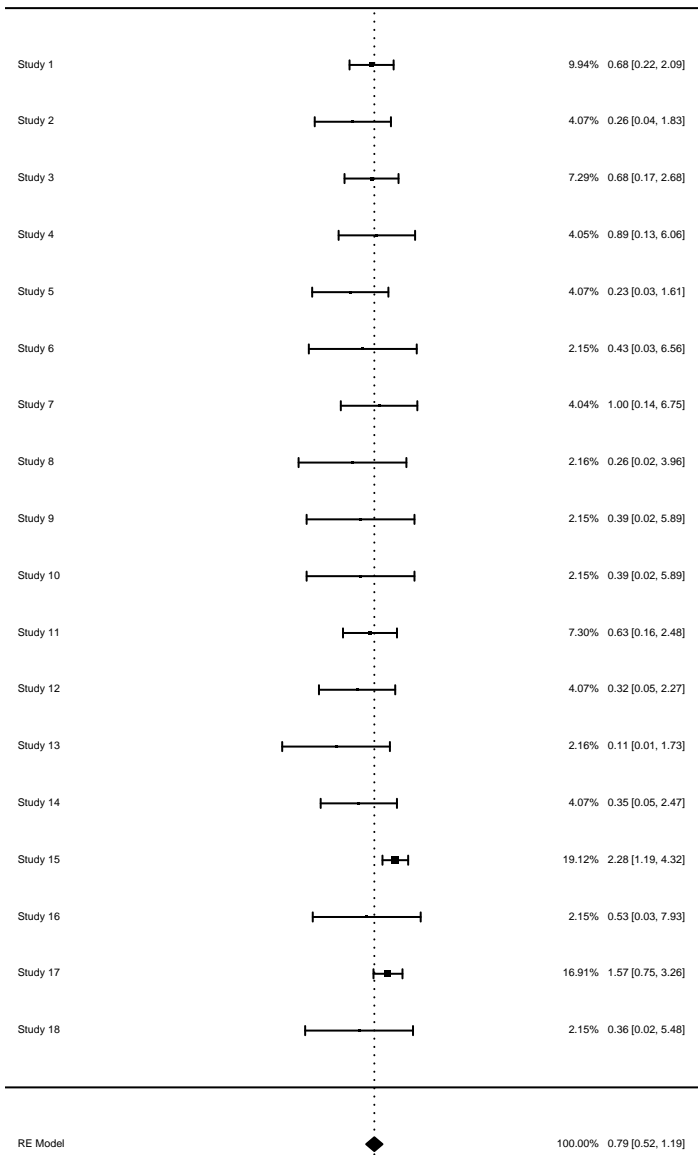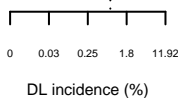

POPF related POMR  
(group C)

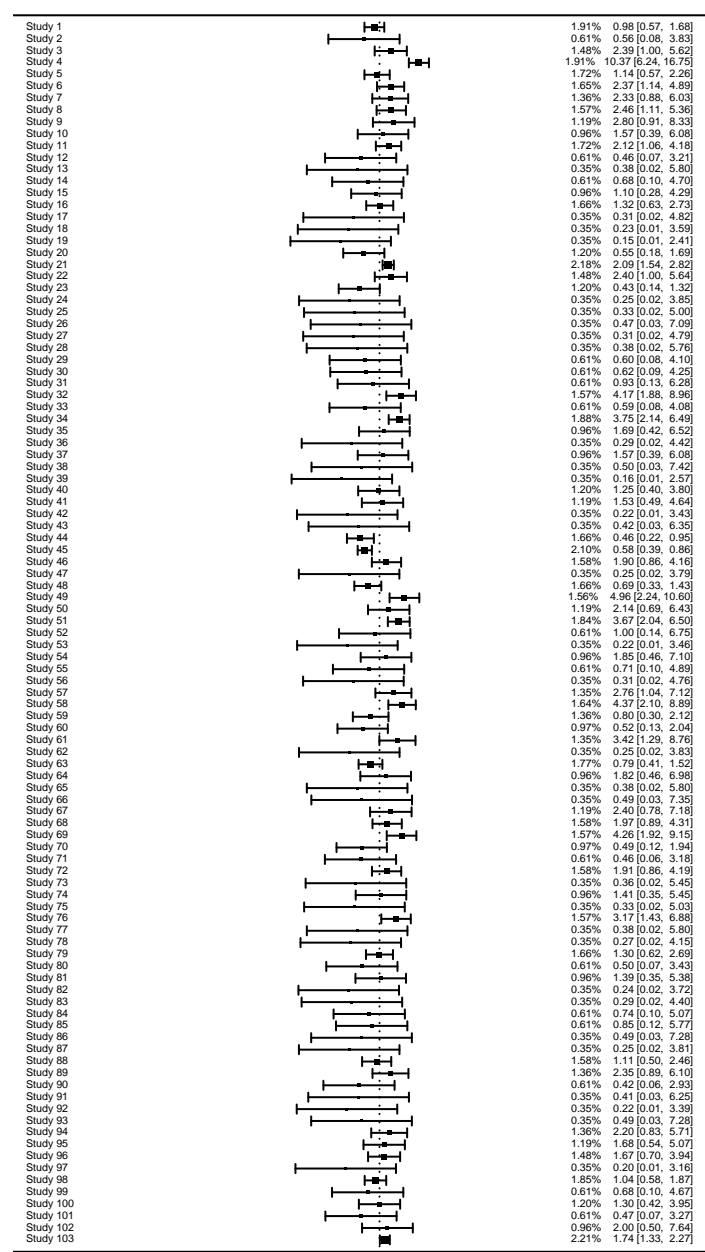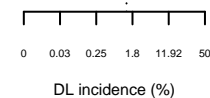

Supplement: Supplementary file 4 — (PDF 23 kb). [file 423_2020_2005_MOESM4_ESM.pdf]

CR-POPF: % POMR  
(group A1-B1)

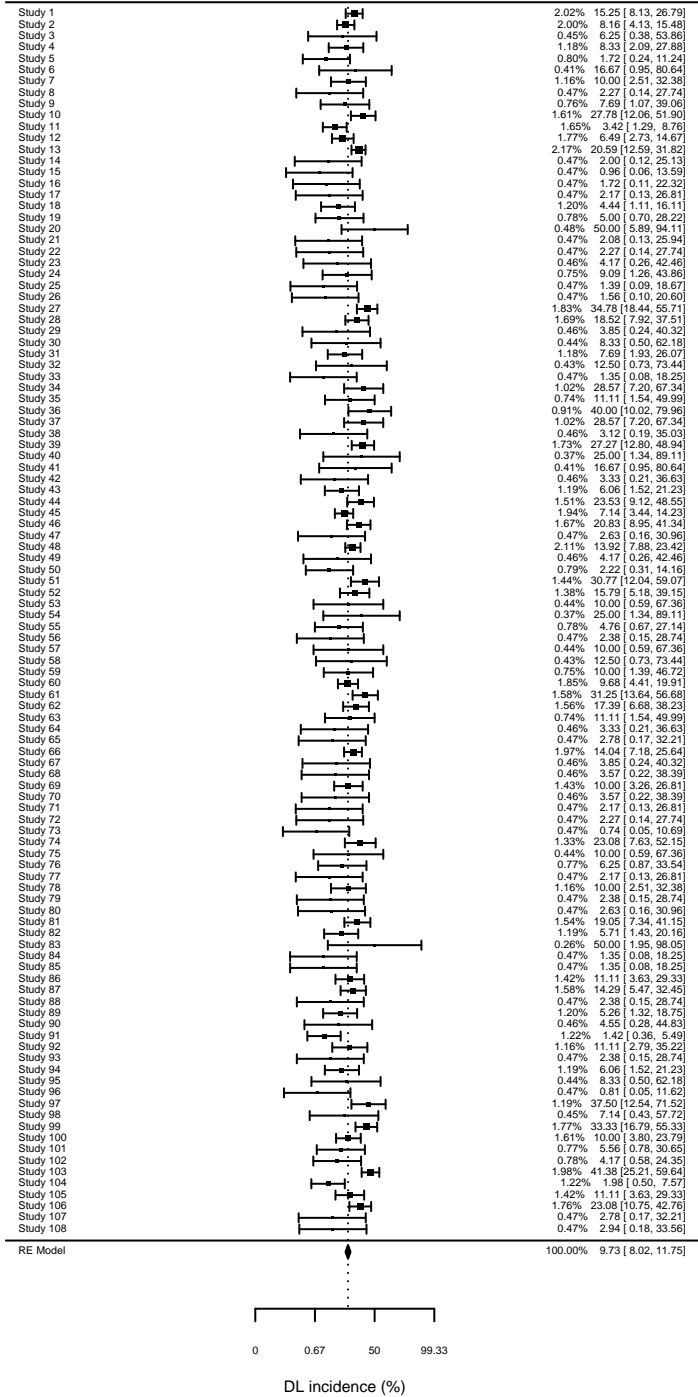

CR-POPF: % POMR  
(group A2-B2)

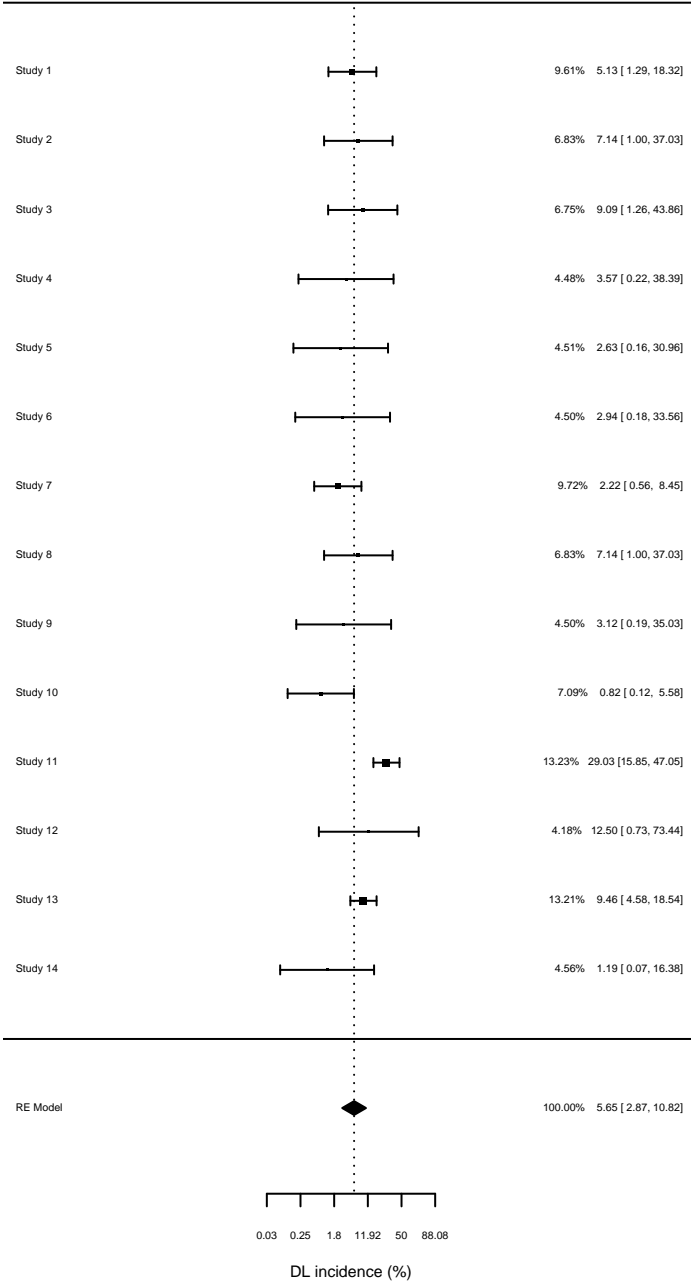

CR-POPF: % POMR  
(group C)

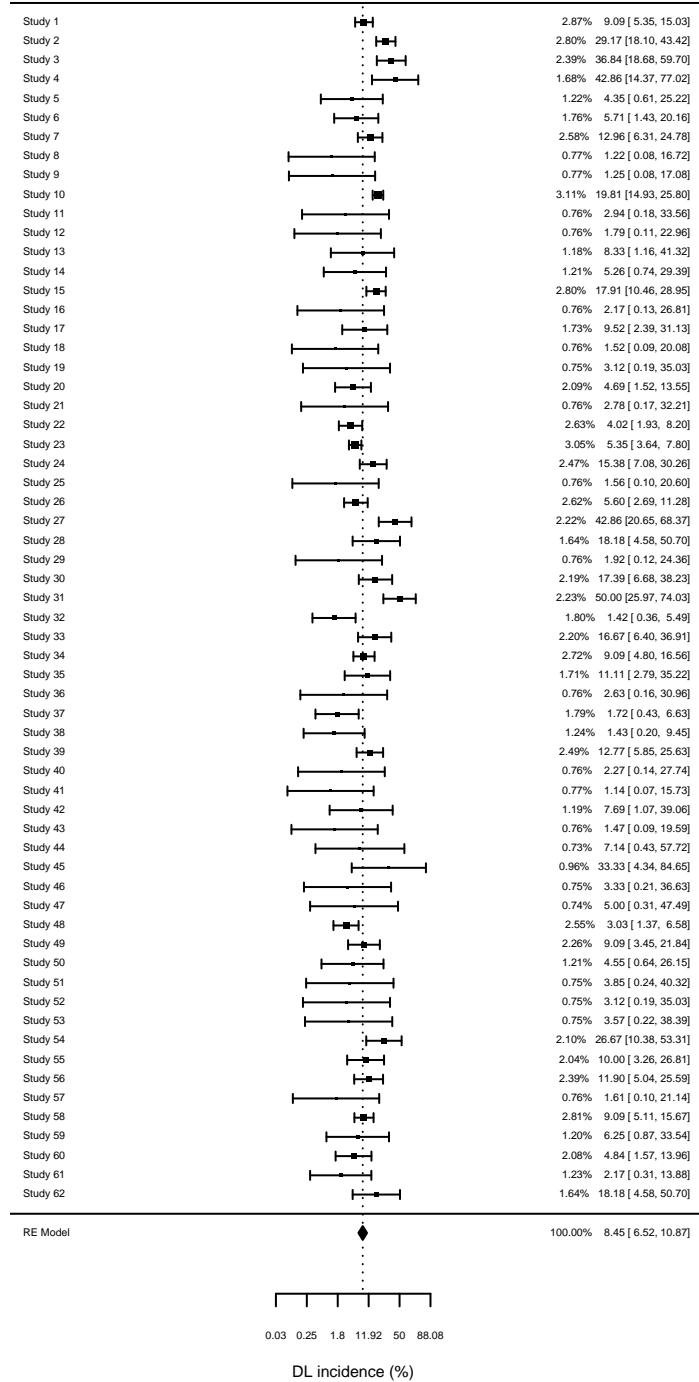

Supplement: Supplementary file 5 — (PDF 18 kb). [file 423_2020_2005_MOESM5_ESM.pdf]

Interventions  
(group A1)

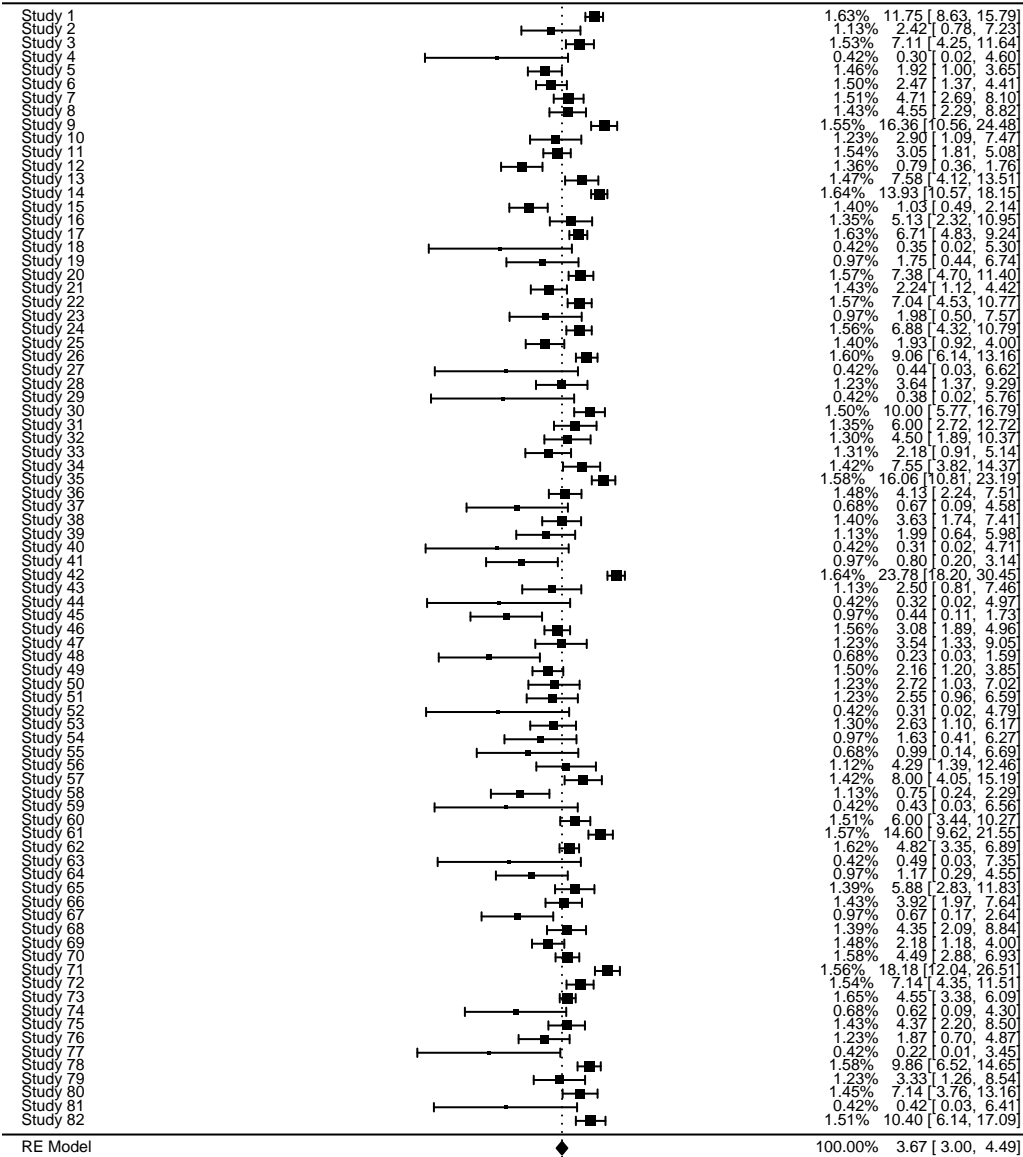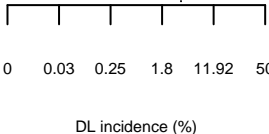

Interventions  
(group A2)

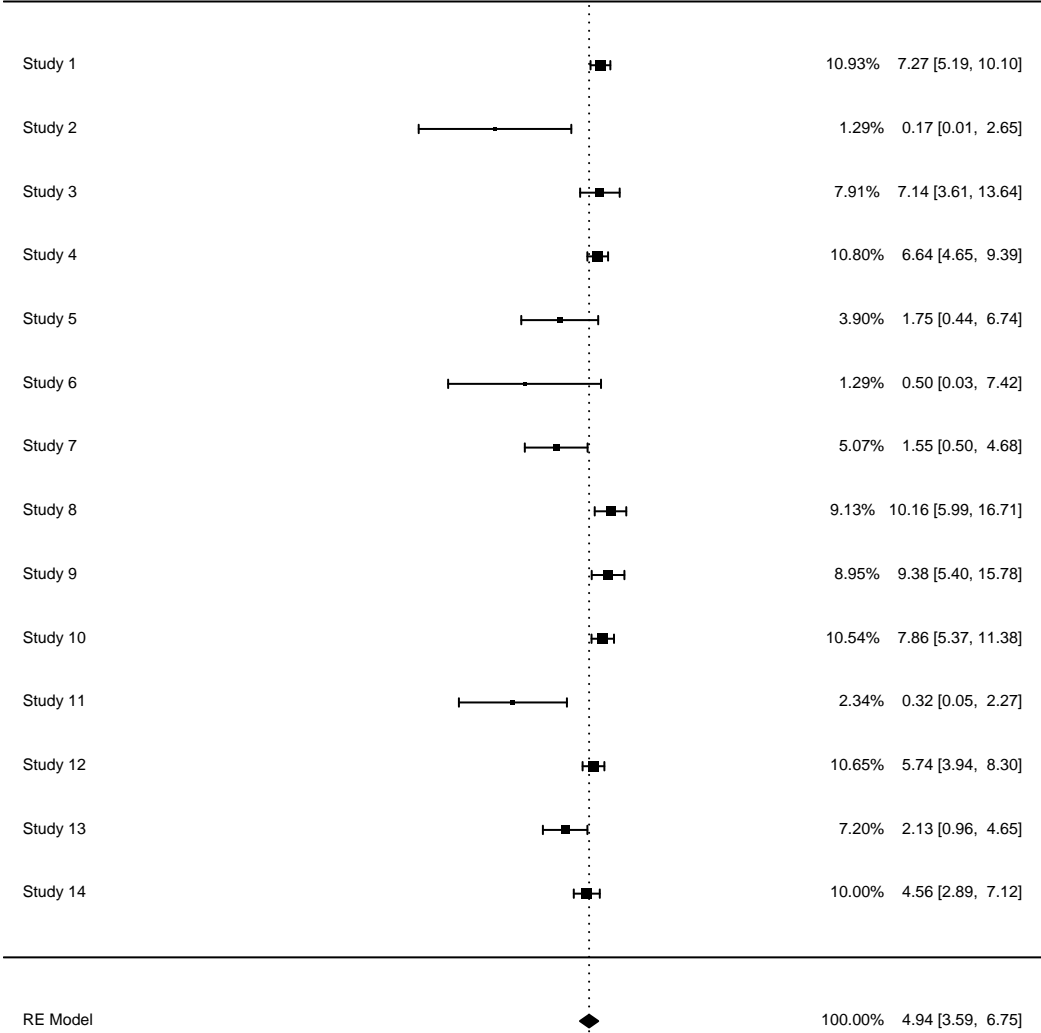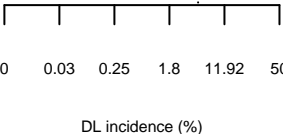

Supplement: Supplementary file 6 — (PDF 11 kb). [file 423_2020_2005_MOESM6_ESM.pdf]

Reoperation rate  
(group A1)

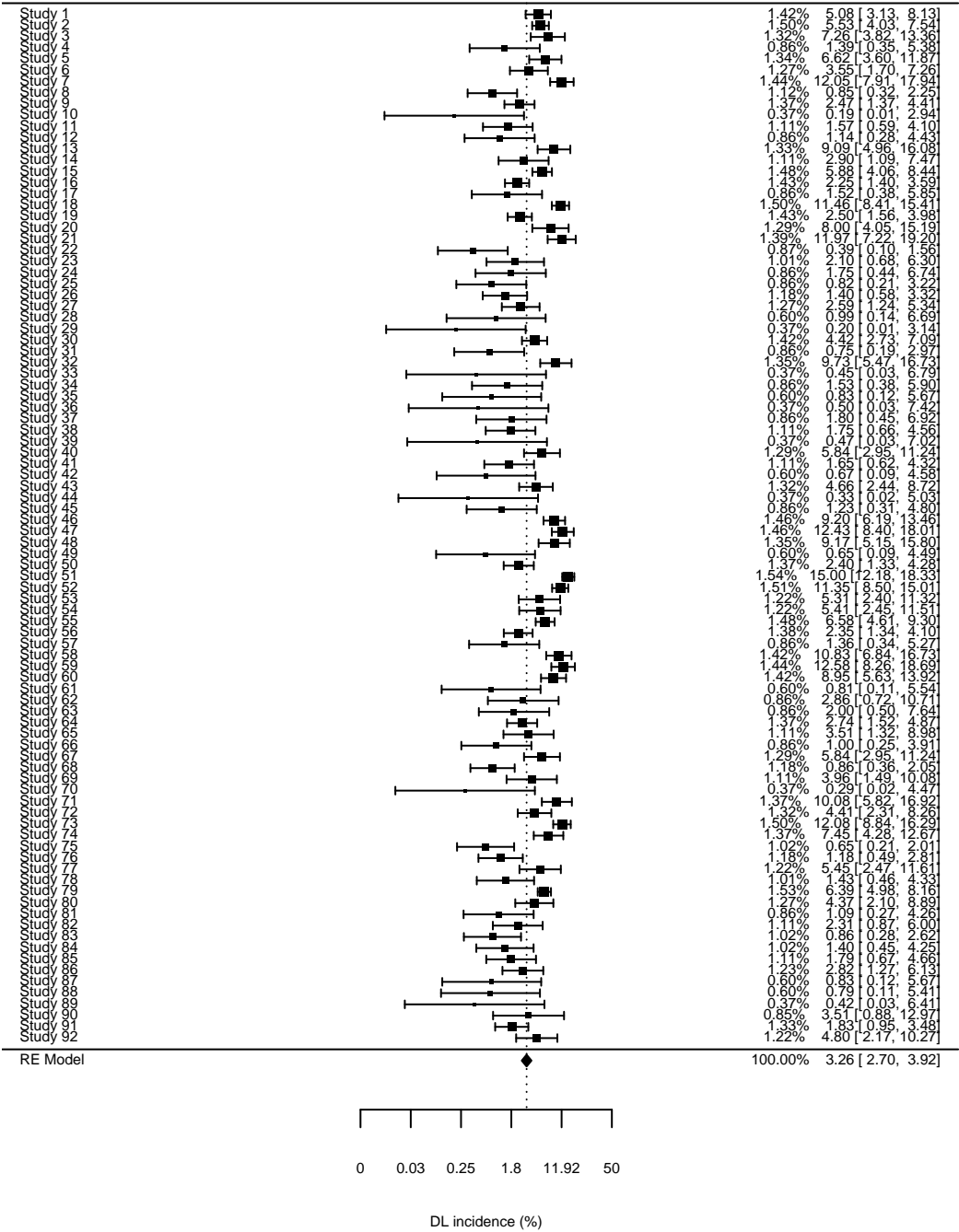

Reoperation rate  
(group A2)

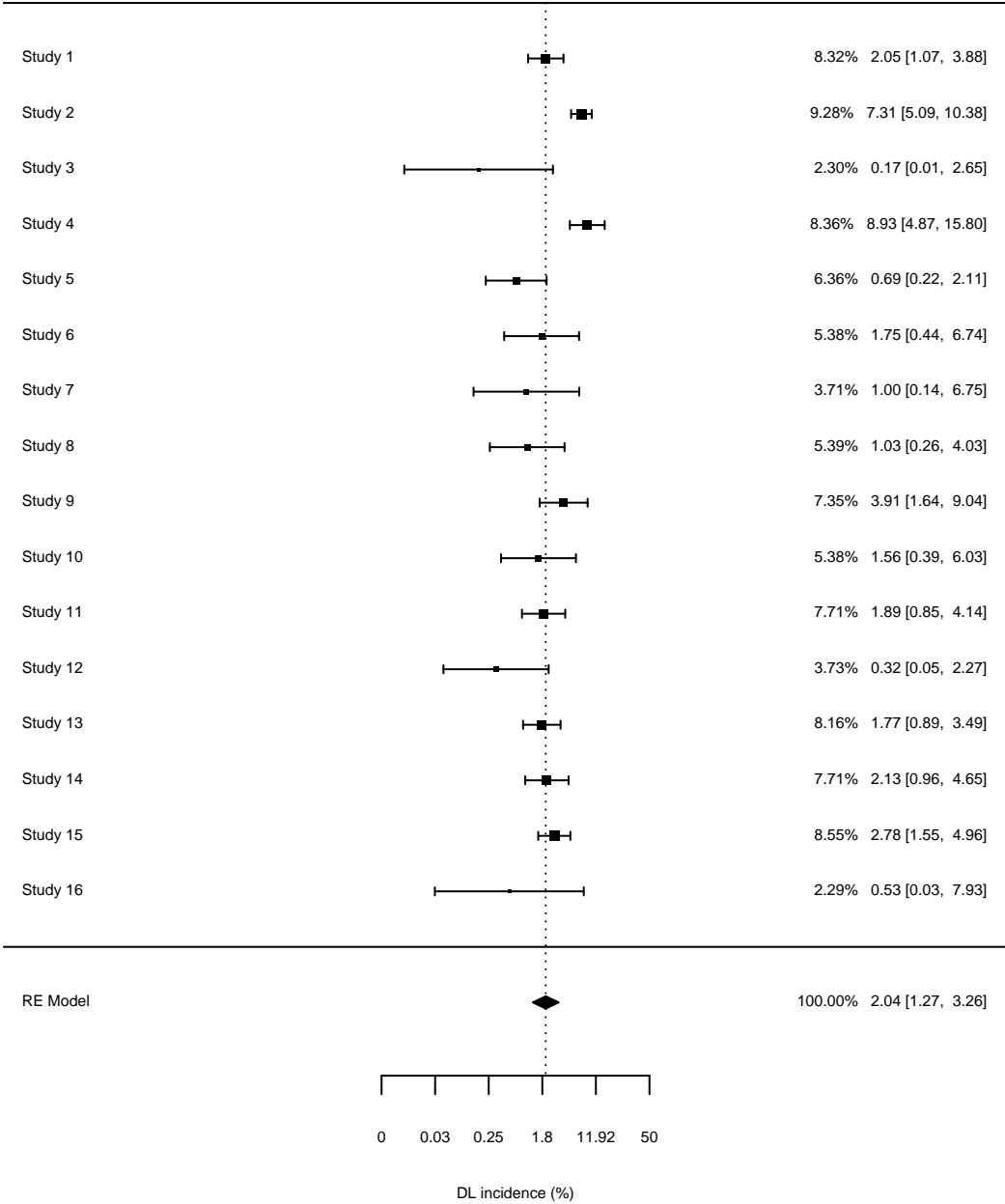

Supplement: Supplementary file 7 — (PDF 12 kb). [file 423_2020_2005_MOESM7_ESM.pdf]

Completion pancreatectomy  
(group A1)

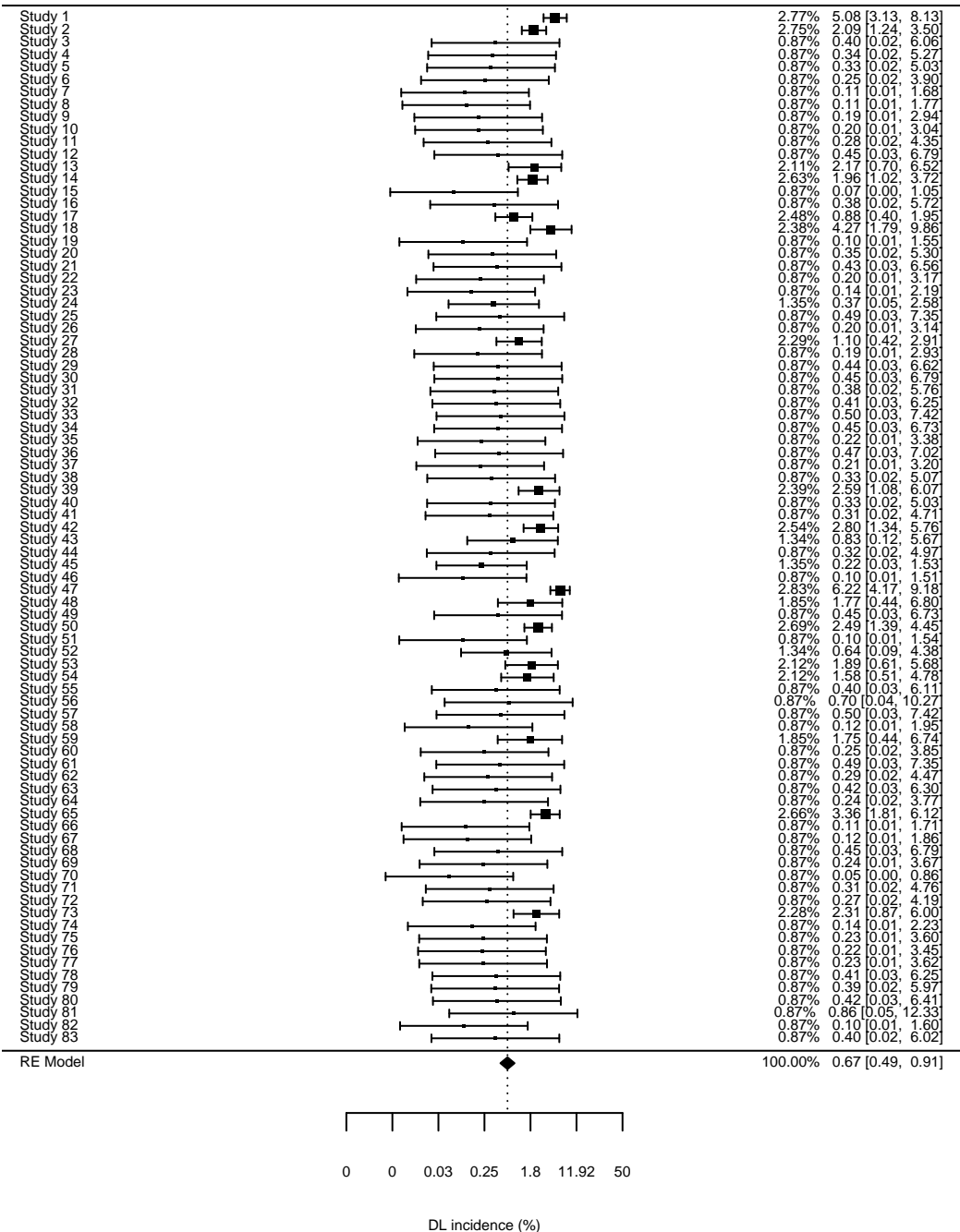

Completion pancreatectomy  
(group A2)

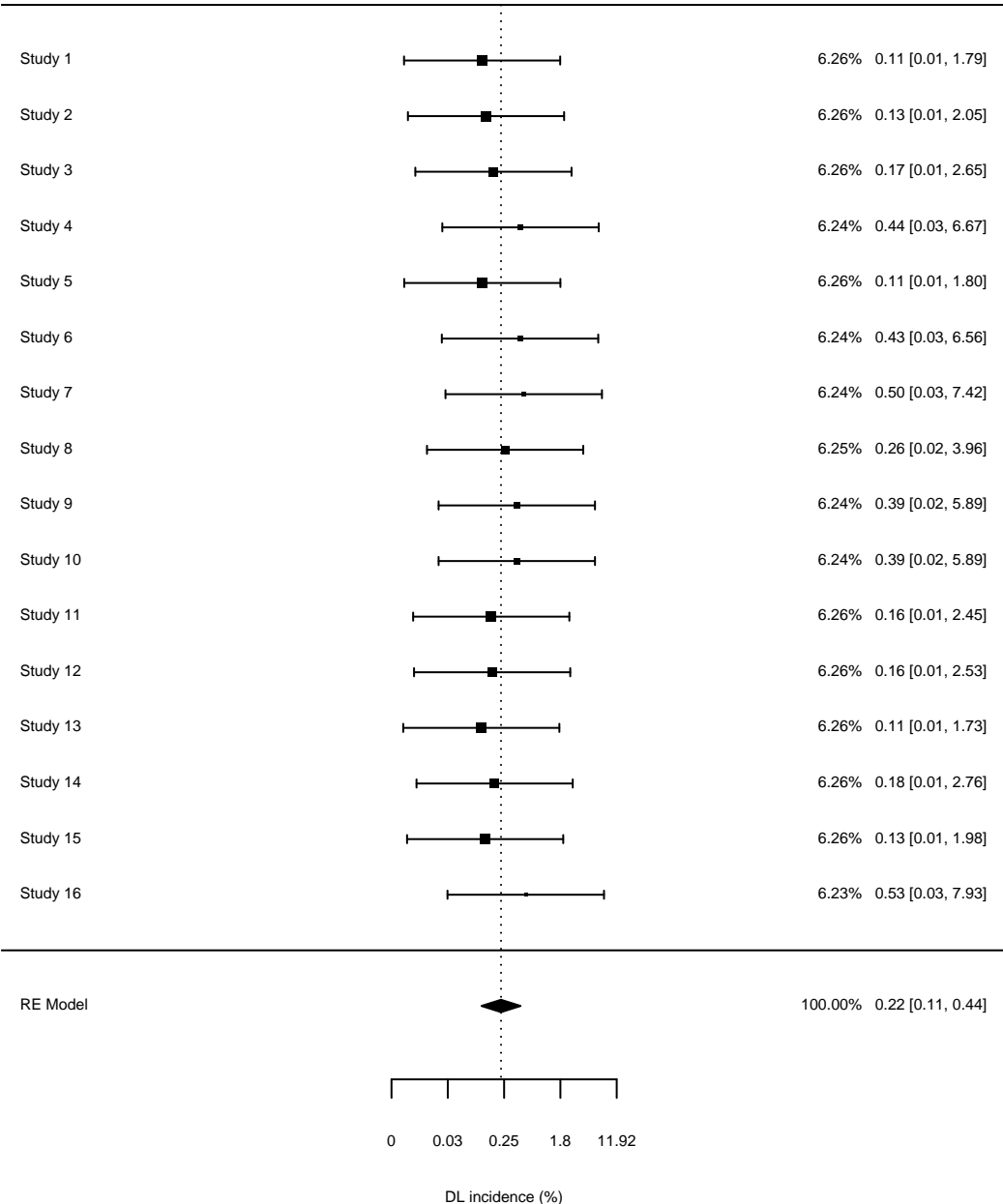

Supplement: Supplementary file 8 — (PDF 11 kb). [file 423_2020_2005_MOESM8_ESM.pdf]

CR-POPF  
(group A1-B1)

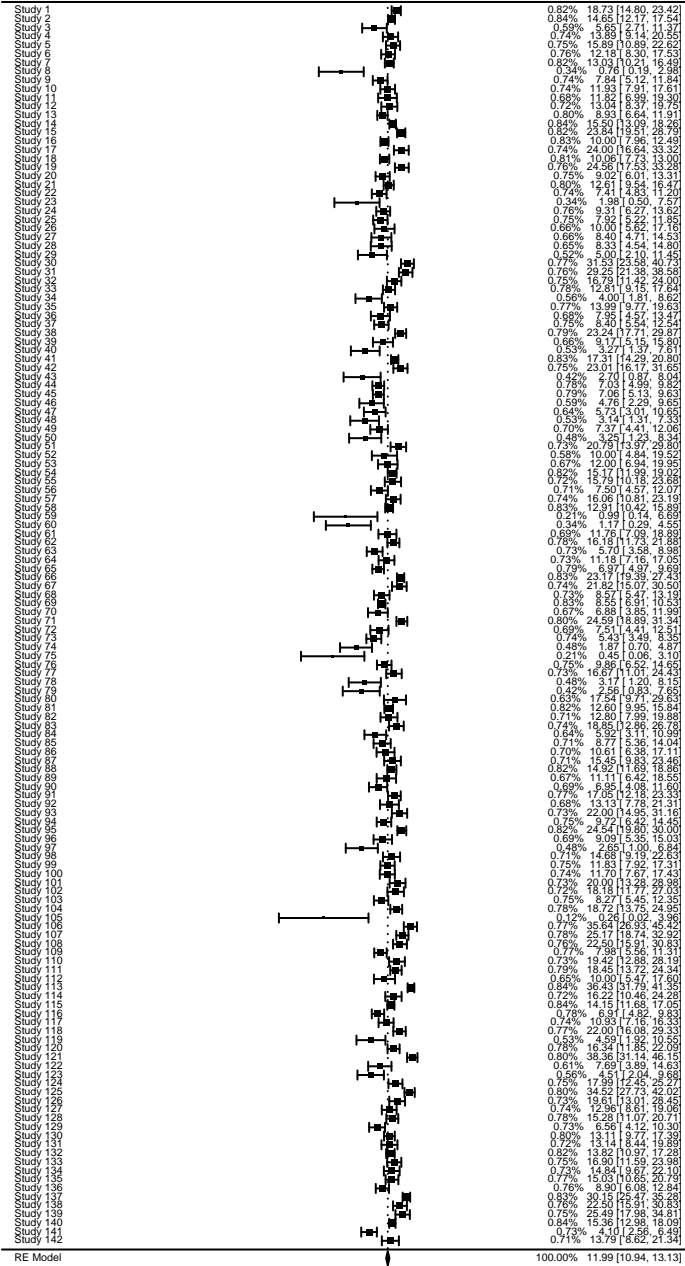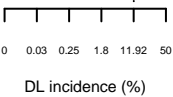

CR-POPF  
(group A2-B2)

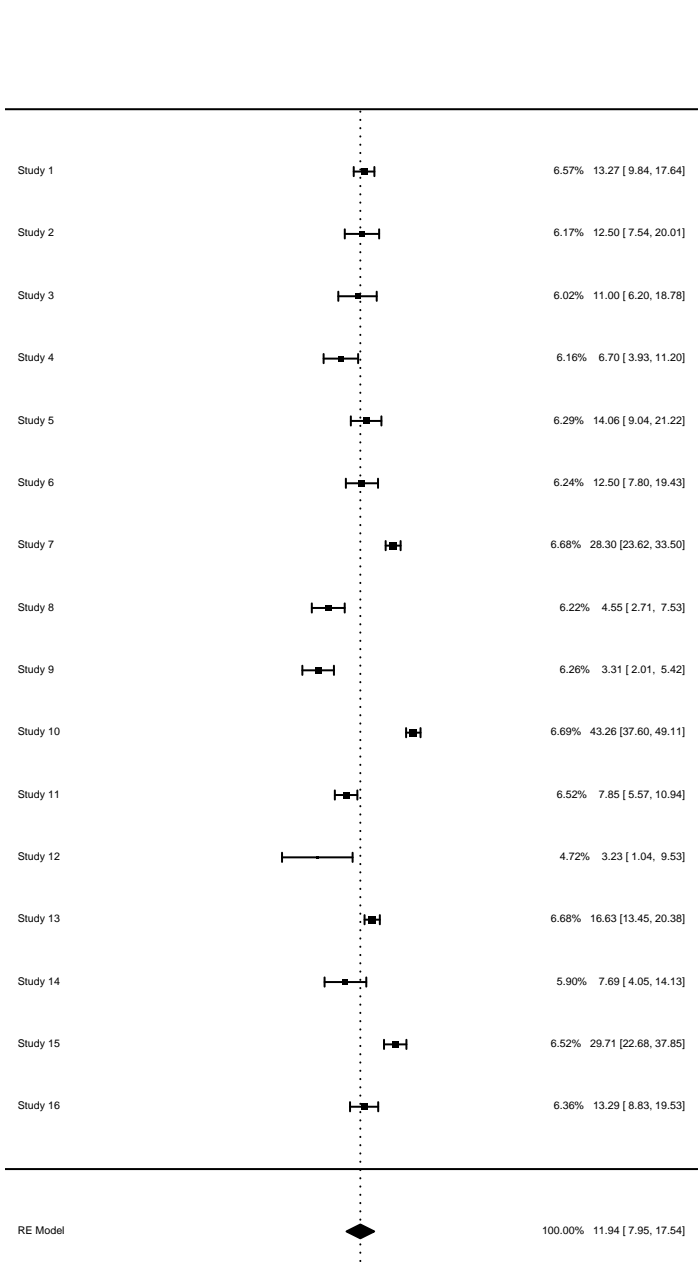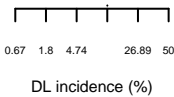

CR-POPF  
(group C)

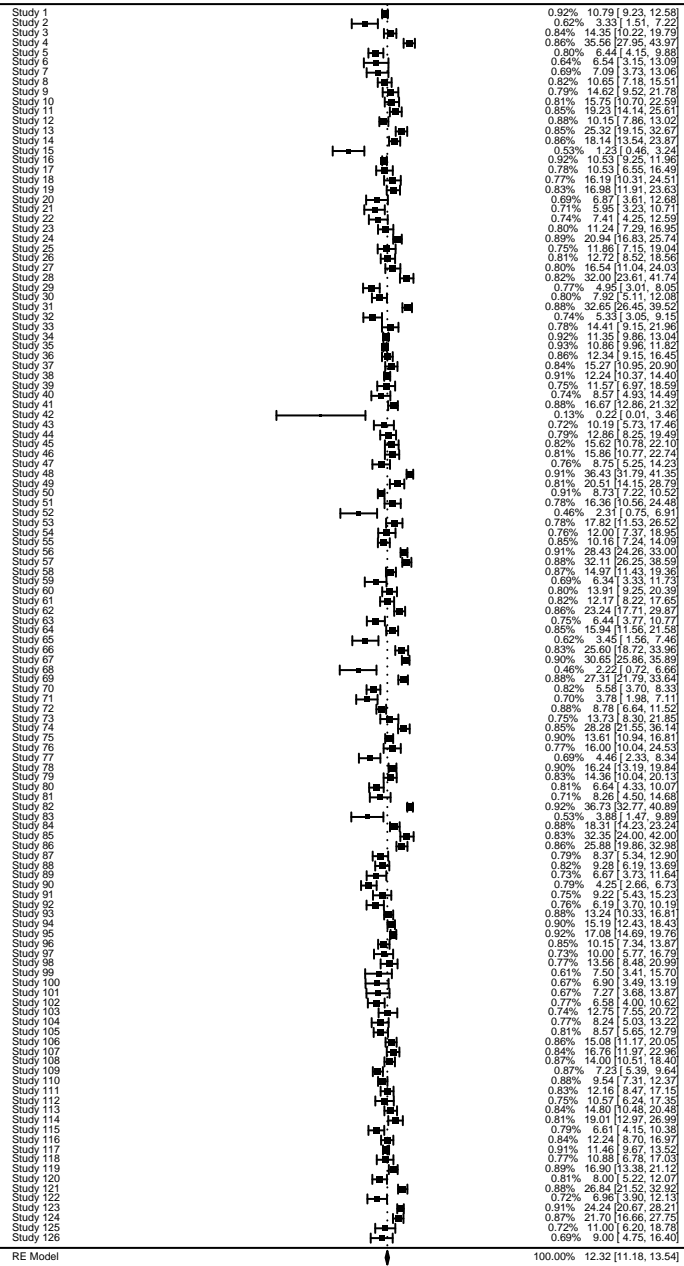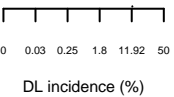

Supplement: Supplementary file 9 — (PDF 25 kb). [file 423_2020_2005_MOESM9_ESM.pdf]

CR-POPF: % POMR  
(group A1-B1)

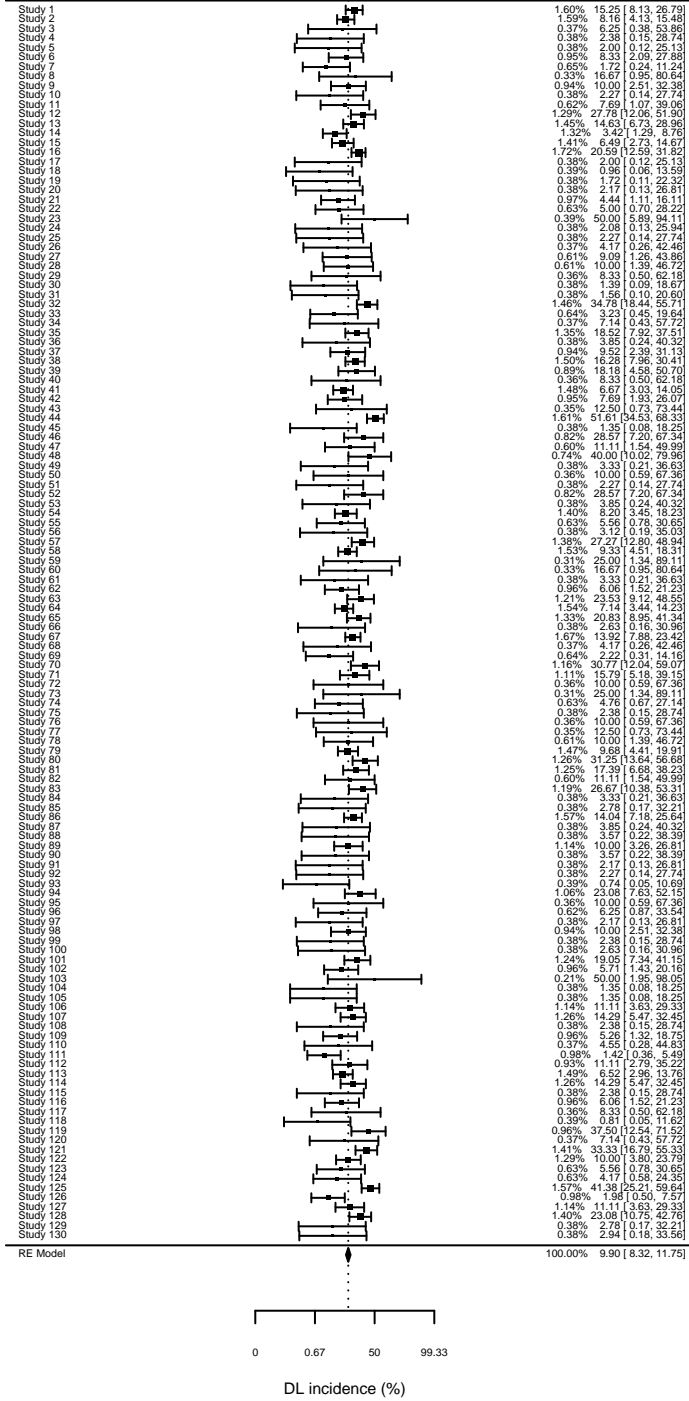

CR-POPF: % POMR  
(group A2-B2)

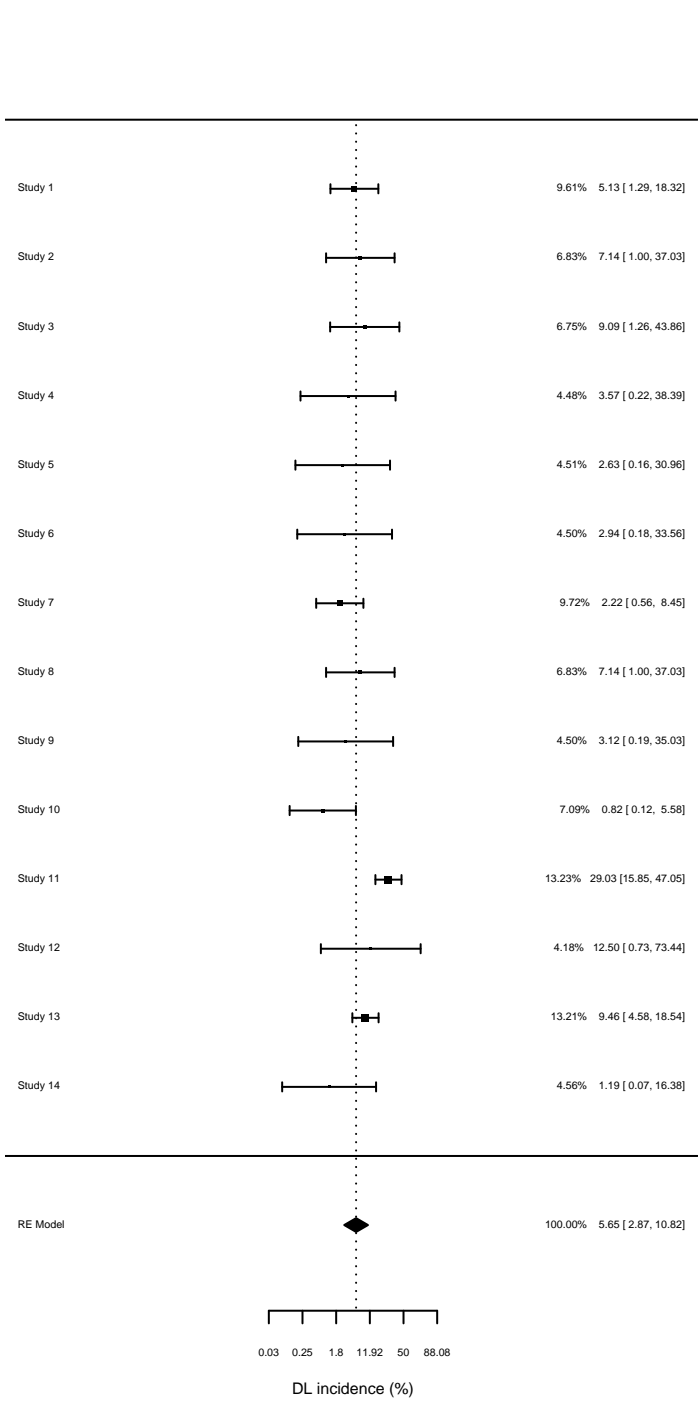

CR-POPF: % POMR  
(group C)

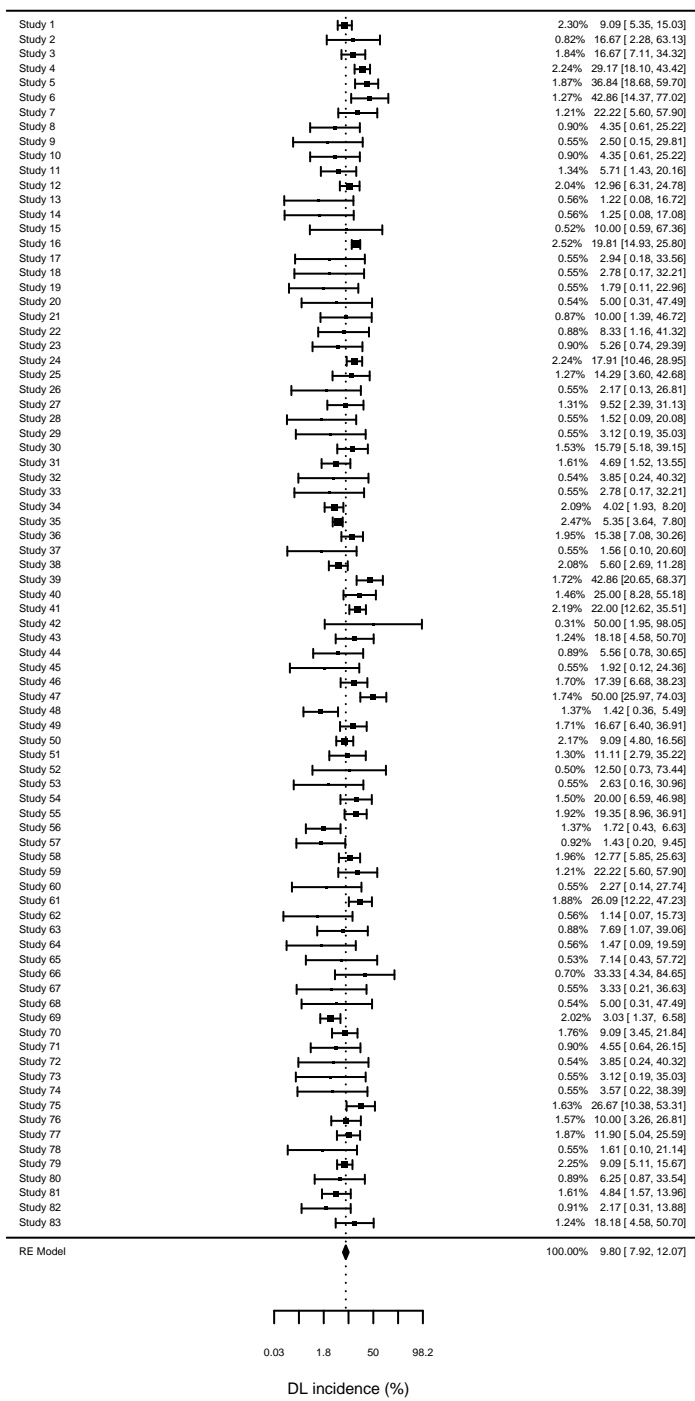

Supplement: Supplementary file 10 — (PDF 20 kb). [file 423_2020_2005_MOESM10_ESM.pdf]
