# Supplementary material for: Systematic review and meta-analysis of surgical drain management after the diagnosis of postoperative pancreatic fistula after pancreaticoduodenectomy: draining-tract-targeted works better than standard management
Source: Langenbecks Arch Surg. 2020 Oct 26;405(8):1219–31. doi: 10.1007/s00423-020-02005-8 (PMC7686010; doi:10.1007/s00423-020-02005-8)
Supplement: Supplementary file 11 — (DOCX 51 kb). [file 423_2020_2005_MOESM11_ESM.docx]

Table 4: Results for primary endpoints in Groups A1-B1, A2-B2, C, and the sample overall.

| Primary endpoints | Group A1-B1 | Group A2-B2 | Group C | Overall | P |
| --- | --- | --- | --- | --- | --- |
| CR-POPF^a^  Studies  Patients  Mean  SD^b^  Average incidence (%)  SD^b^  Pooled incidence (%)  DL incidence^c^ (%) | 142/159  31,669  223.02  157.15  13.04  7.80  12.71  12.50 | 16/20  3,663  228.94  126.99  14.24  10.89  14.50  13.95 | 126/148  38,953  309.15  454.91  14.46  7.90  13.00  13.08 | 284/327^e^  74,285  261.57  326.16  13.30  8.02  12.95  12.83 | =0.009  =0.699 |
| CR-POPF^a^-related mortality  Studies  Patients with CR-POPF  Mean  SD^b^  Average incidence (%)  SD^b^  Pooled incidence (%)  DL incidence (%) | 130/159  3,564  27.42  25.39  8.41  11.48  7.97  5.75 | 14/20  501  35.79  35.29  5.00  7.84  4.79  3.48 | 83/148  3,535  42.59  63.83  9.84  11.49  8.12  6.24 | 227/327*  7,600  33.48  44.38  8.72  11.31  7.83  5.76 | =0.031  =0.090 |

^a^ All patients with CR-POPF (both those defined on the basis of the ISGPF/ISGPS classifications [32,33] and the symptomatic ones reported before the publication of the ISGPF criteria) were considered

^b^ Standard deviation

^c^ DL incidence (%): DerSimonian-Laird estimator

^e^ Patients in one study were shared between *Subgroups* A1 and A2 depending on their postoperative surgical drain management
